# Cell-impermeable staurosporine analog targets extracellular kinases to inhibit HSV and SARS-CoV-2

**DOI:** 10.1038/s42003-022-04067-4

**Published:** 2022-10-16

**Authors:** Natalia Cheshenko, Jeffrey B. Bonanno, Hans-Heinrich Hoffmann, Rohit K. Jangra, Kartik Chandran, Charles M. Rice, Steven C. Almo, Betsy C. Herold

**Affiliations:** 1 Department of Pediatrics, Albert Einstein College of Medicine, Bronx, NY USA; 2 Department of Biochemistry, Albert Einstein College of Medicine, Bronx, NY USA; 3 Laboratory of Virology and Infectious Disease, The Rockefeller University, New York, NY USA; 4 Department of Microbiology and Immunology, Albert Einstein College of Medicine, Bronx, NY USA; 5 Present Address: Department of Microbiology and Immunology, Louisiana State University Health Science Center-Shreveport, Shreveport, LA USA

**Keywords:** Herpes virus, Kinases

## Abstract

Herpes simplex virus (HSV) receptor engagement activates phospholipid scramblase triggering Akt translocation to the outer leaflet of the plasma membrane where its subsequent phosphorylation promotes viral entry. We hypothesize that this previously unrecognized outside-inside signaling pathway is employed by other viruses and that cell-impermeable kinase inhibitors could provide novel antivirals. We synthesized a cell-impermeable analog of staurosporine, CIMSS, which inhibited outer membrane HSV-induced Akt phosphorylation and blocked viral entry without inducing apoptosis. CIMSS also blocked the phosphorylation of 3-phosphoinositide dependent protein kinase 1 and phospholipase C gamma, which were both detected at the outer leaflet following HSV exposure. Moreover, vesicular stomatitis virus pseudotyped with SARS-CoV-2 spike protein (VSV-S), but not native VSV or VSV pseudotyped with Ebola virus glycoprotein, triggered this scramblase-Akt outer membrane signaling pathway. VSV-S and native SARS-CoV-2 infection were inhibited by CIMSS. Thus, CIMSS uncovered unique extracellular kinase processes linked to HSV and SARS-CoV-2 entry.

## Abstract (web summary)

A cell-impermeable analog of staurosporine blocks phosphorylation events at the outer leaflet of the plasma membrane, which prevents herpes simplex virus and SARS-CoV-2 entry without inducing apoptosis.

## Introduction

Herpes simplex virus serotypes 1 and 2 (HSV-1 and HSV-2) are major global health problems, representing leading causes of infectious corneal blindness, sporadic fatal encephalitis and significant perinatal disease. The public health impact of HSV-2, in particular, is magnified because it is a key coinfection fueling the HIV epidemic^1^. The epidemiology of HSV highlights the need to identify novel strategies for treatment. The molecular complexity of HSV entry has impeded the development of antivirals targeting this process. We previously demonstrated that HSV enters most human epithelial cells through a complex calcium-dependent signaling pathway^2–4^. Specifically, binding of HSV envelope glycoprotein C (gC) (HSV-1) or glycoprotein B (gB) (HSV-2) to cellular heparan sulfate proteoglycans and engagement between glycoprotein D (gD) and a cellular receptor, most commonly nectin-1, triggers intracellular calcium ion (Ca^2+^) transients near the plasma membrane. These transients activate phospholipid scramblase 1 (PLSCR1), a Ca^2+^-responsive enzyme responsible for the bidirectional translocation of phospholipids, including phosphatidylserine (PtdS), between the inner and outer leaflets of the plasma membrane. Notably, we found that these lipid movements are also associated with the translocation of Akt to the outer leaflet of the plasma membrane where it is phosphorylated by yet to be determined kinases^2,5^. Extracellular phosphorylation of Akt is associated with downstream signaling events, perhaps involving outside-in signaling, which culminate in HSV entry^3–6^. Small interfering RNA (siRNA) targeting PLSCR1 or Akt and pharmacological kinase inhibitors prevented HSV entry and infection^2,4^. It is notable that prolonged PtdS exposure on the outer leaflet of the plasma membrane is an apoptotic signal, which would be detrimental to viral infection and propogation^7^. HSV circumvents this liability by orchestrating the translocation of PtdS back to the inner leaflet within 4 h of viral exposure via a process that is also dependent on PLSCR1 and viral glycoprotein L (gL)^2^.

Translocation of PtdS to the outer leaflet of the plasma membrane is a well-described phenomenon in cell biology, but the observation that the protein kinase Akt, which typically shuttles between the cytosol and the inner leaflet of the plasma membrane, also becomes accessible on the outer leaflet had not been previously appreciated. This translocation of Akt to the outer leaflet of the plasma membrane may be employed by other viruses and biological processes associated with scramblase activation, as evidenced by our findings that ionomycin, a calcium ionophore, also activates PLSCR1 and is associated with externalization and subsequent phosphorylation of Akt^2^. These observations suggested that cell-impermeable inhibitors that selectively target extracellular kinase activities could be developed as tool compounds to study outside-inside signaling and as novel anti-viral drugs. As a proof of concept, we modified staurosporine, a non-specific ATP-competitive pan-kinase inhibitor that blocks Akt phosphorylation and triggers cellular apoptosis, to generate a cell-impermeable analog. This cell-impermeable staurosporine analog (CIMSS) prevented HSV-induced phosphorylation of Akt at the outer leaflet of the plasma membrane and blocked subsequent HSV entry without inducing apoptosis or inhibiting intracellular Akt phosphorylation in response, for example, to insulin. Antibodies to Akt also inhibited HSV entry. CIMSS also blocked the extracellular phosphorylation of 3-phosphoinositide dependent protein kinase 1 (PDPK1) and phospholipase C gamma (PLCγ), which were also detected at the outer leaflet of the plasma membrane in response to HSV infection. Using CIMSS as a tool to identify other viruses that might exploit a similar signaling pathway, we found that vesicular stomatitis virus (VSV) pseudotyped with SARS-CoV-2 spike protein (VSV-S), but not native VSV or VSV-pseudotyped with the Ebola virus glycoprotein (VSV-EBOV GP) triggered transloction to, and phosphorylation of Akt, PDPK1 and PLCγ at the outer leaflet of the plasma membrane. CIMSS blocked Akt phosphorylation in response to VSV-S and inhibited both VSV-S and native SARS-CoV-2 infection.

## Results

### Design, synthesis and characterization of a cell-impermeable staurosporine analog

Hypothesizing that inhibition of Akt and other upstream or downstream activating kinases that have been translocated to the outer leaflet of the plasma membrane would modulate viral entry, we selected the well studied broad spectrum kinase inhibitor staurosporine as a scaffold to develop an inhibitor that would selectively target extracellular kinases^8^. As there was no extant structure of an Akt:staurosporine complex, we generated a model by overlaying the coordinates of Akt1 (one of three Akt isoforms) bound to a small molecule inhibitor (Protein Data Bank (PDB) entry 3MVH) with those of the related ribosomal protein S6 kinase beta-1, p70S6K1, bound to staurosporine (~46% sequence identity; PDB entry 3A60). The structural alignment revealed close agreement between the protein coordinates (RMSD = 1.0 Å for 211 aligned Cα pairs). The 2° amine moiety of staurosporine appeared to be solvent accessible and unhindered in the resulting model and thus represented a candidate for synthetic elaboration to introduce a solvent exposed polar group, which would impair cell entry while maintaining Akt binding and inhibitory properties.

**Fig. 1:**
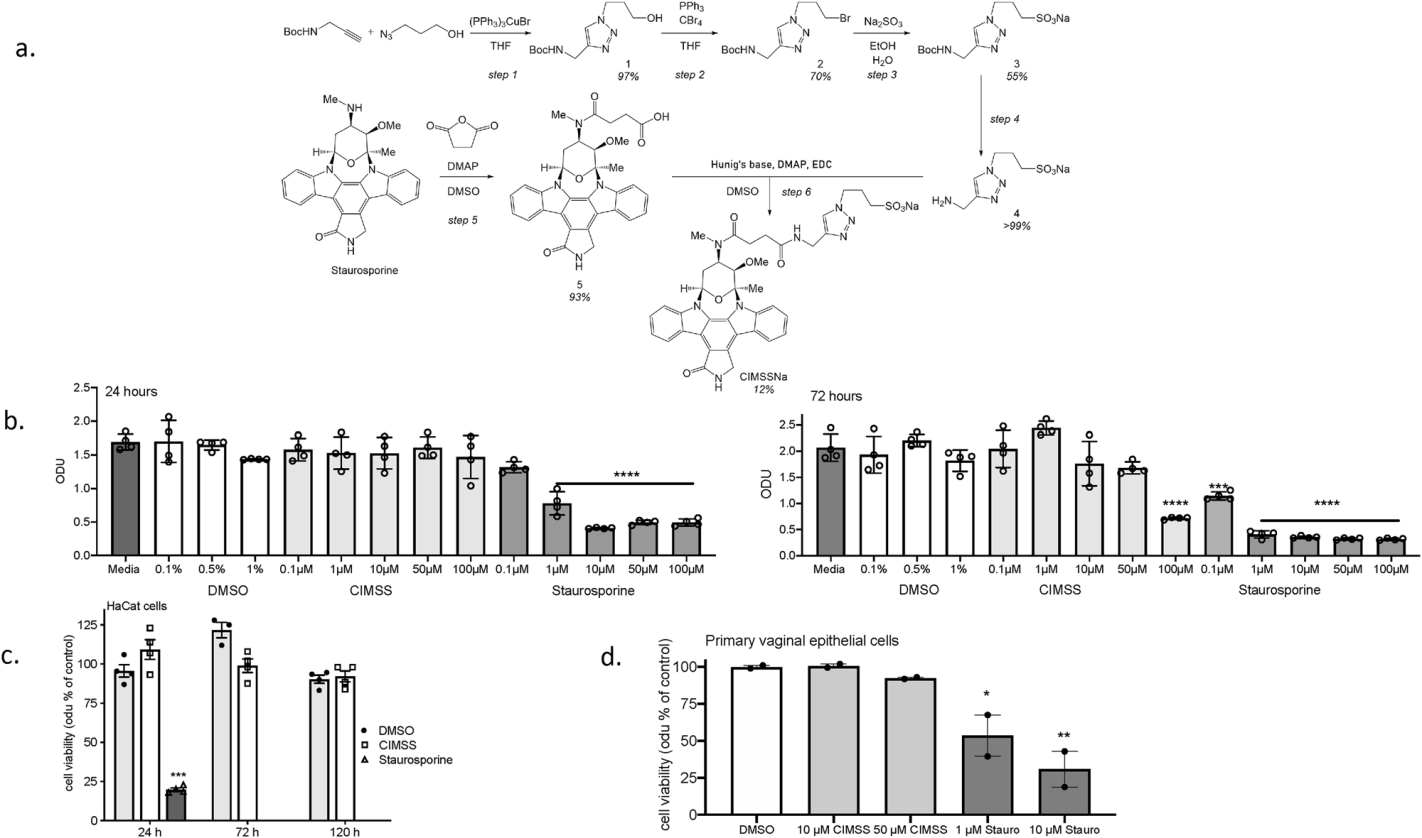
Synthesis of cell impermeable staurosporine analog (CIMSS) and the effects of the parental drug and the analog on cell proliferation and viability. **a** Scheme illustrating the synthetic route to the sodium salt of CIMSS: the secondary amine of staurosporine was derivatized via amidation of succinic anhydride and the resulting carboxylate (compound 5) was condensed with aminomethyl-triazol-propane-sulfonate (compound 4, which was obtained in four steps from NBoc-propargylamine and azidopropanol) to afford CIMSSNa in modest yield as a white powder. **b** HaCat cells (~50% confluence) were cultured in media containing increasing concentrations of staurosporine or CIMSS (0.1–100 μM) or the equivalent concentration of DMSO (0.1, 0.5, or 1%) and cell proliferation and viability (optical densitometry units, odu) quantified after 24 and 72 h. **c** Confluent HaCat cells were cultured in media containing 10 μM CIMSS, 10 μM staurosporine or 0.1% DMSO (control) and viability assessed after 24, 72 and 120 h. Note that staurosporine was completely cytotoxic after 72 and 120 h of exposure and thus no bar is visible. **d** Primary vaginal epithelial cells (~50% confluent) were cultured in media containing 0.5% DMSO, 10 or 50 μM CIMSS or 1 or 10 μM staurosporine for 120 h and cell proliferation and viability monitored. Results are presented as mean ± SEM odu as a percentage of the DMSO control (*n* = 2 independent experiments each conducted in duplicate for **c** and 1 experiment conducted in duplicate for **d**). Results in **b**–**d** were compared by ANOVA, *p < 0.05, **p < 0.01, ***p < 0.001, ****p < 0.0001).

Based on this modeling, we devised a synthetic scheme to install a moiety bearing a sulfonate functionality at the 2° amine of staurosporine, yielding a cell-impermeable analogue of staurosporine (Fig. 1a). We hypothesized that CIMSS would inhibit HSV entry without inducing apoptosis or interfering with intracellular Akt signaling pathways triggered by other stimuli such as activation of insulin receptors^9^. In an in vitro kinase inhibitor assay performed against a panel of 393 kinases with 10 μM ATP, CIMSS (10 μM) retained the pleiotropic inhibitory property of staurosporine including inhibition of all three Akt isoforms (Akt1, Akt2, Akt3) and PDPK1, the enzyme responsible for cytoplasmic phosphorylation and activation of Akt isoforms, by >75% (Supplementary Data 1). Similar levels of inhibition were observed for a range of Src family kinases, Aurora kinases, JAK kinases and receptor tyrosine kinases. The 50% inhibitory concentration (IC_50_) for CIMSS and staurosporine were comparable for PDPK1, for example, but CIMSS was less effective than staurosporine for other kinases, including insulin receptor, Akt1 and PKA (~40, ~230, and ~46-fold less effective; Supplementary Table 1). The altered inhibitory profile of CIMSS is consistent with a previous report^10^, in which acylation of the 4’-methylamine negatively impacted IC_50_s, with increases of 1–2 orders of magnitude for diverse kinases, including PIM1 (>200-fold), CHK1 (84-fold), CDK2 (50-fold), and PKA (14-fold).

The low permeability of CIMSS compared to control compounds was demonstrated by measuring the apparent permeability coefficient (Papp) with confluent MDCK cells as well as in an artifical membrane permeability assay (PAMPA) (Supplementary Table 2). This behavior was further assessed in cell viability studies, which demonstrated that unmodified staurosporine significantly inhibited HaCat (human keratinocyte) cell growth at concentrations as low as 1 μM at 24 h (h), and 0.1 μM at 72 h, while no significant inhibition was observed with CIMSS except when cells were cultured for 72 h in media containing 100 μM CIMSS (Fig. 1b). Similarly, little or no cytotoxicity was observed when fully confluent HaCat cells were incubated with 10 μM CIMSS or when ~50% confluent primary vaginal epithelial cells were cultured with 10 or 50 μM CIMSS for up to 120 h (Fig. 1c, d).

**Fig. 2:**
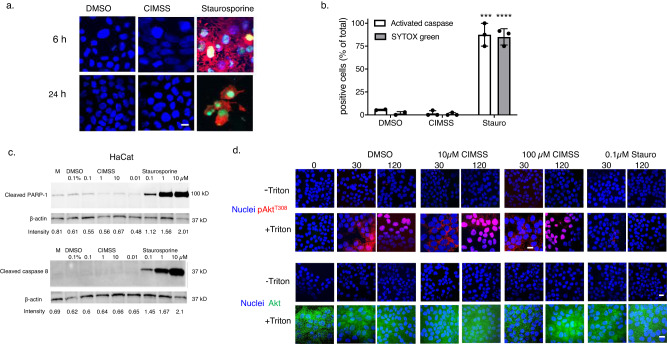
CIMSS does not induce apoptosis or block the intracellular phosphorylation of Akt in response to insulin. HaCat cells were exposed to 0.1% DMSO, 10 µM CIMSS, or 10 µM staurosporine and after 6 or 24 h of incubation, the cells were fixed and stained for activated caspases (red), integrity of plasma membrane with SYTOX Green, and nuclei (Hoechst stain, blue). **a** Representative images taken with ZeissLive/DuoScan (objective 100×1.4, bar = 10 μm) and **b** the percentage of cells positive for activated caspase and SYTOX Green at 24 h was quantified after counting ~100 cells from four independent fields, *n* = 3 experiments; asterisks indicate significance relative to DMSO (unpaired *t*-test, ***p < 0.001; ****p < 0.0001). **c** HaCat cells were exposed to 0.1% DMSO, 0.1, 1, or 10 µM CIMSS or 0.01, 0.1, 1, or 10 µM staurosporine and after 8 h of incubation, lysates were prepared and analyzed by western blotting for cleaved PARP-1 or cleaved caspase 8. The intensity of cleaved PARP-1 or caspase 8 (relative to β-actin) is indicated below each lane. The immunoblot is representative of two independent experiments. **d** HaCat cells were exposed to insulin (10 µM) in the absence or presence of 10 or 100 µM CIMSS or 0.1 µM staurosporine for 30 and 120 min, fixed with or without Triton X-100, stained for nuclei (blue), pAkt^T308^ (red), or total Akt (green). Representative images from two independent experiments obtained with Leica SP8 microscope equipped with objective 63 × 1.4 are shown (bar = 10 μm).

Consistent with its cell impermeability, 10 μM CIMSS did not induce apoptosis when assessed by SYTOX Green and anti-caspase antibody staining (Fig. 2a, b). Similaly, compared to cells treated with 0.1 µM DMSO, CIMSS at doses of 0.1, 1, or 10 µM did not induce PARP-1 or caspase 8 cleavage whereas cleavage of both was observed with staurosporine at concentrations as low as 0.1 µM (Fig. 2c and Supplementary Fig. 1). Furthermore, CIMSS did not block Akt phosphorylation in response to insulin. HaCat cells were treated with 10 μM insulin in the absence or presence of CIMSS (10 or 100 µM) or staurosporine (0.1 µM), fixed with or without Triton X-100 permeabilization and stained with conjugated antibodies to detect phosphorylated Akt (pAkt^t308^) (red) or total Akt (green); nuclei were stained with DAPI (blue). We used higher doses of CIMSS than staurosporine based on the in vitro kinase activity (Supplementary Data 1). Following insulin treatment, Akt was only visualized in permeabilized cells and its intracellular phosphorylation was inhibited by staurosporine at doses as low as 0.1 μM, but not by 100 μM CIMSS (Fig. 2c). These findings indicate that engagement of the insulin receptor by insulin does not trigger translocation of Akt to the outer leaflet of the plasma membrane and that phosphorylation of cytoplasmic Akt is inhibited by staurosporine but is insensitive to CIMSS. These observations, together with direct permeability measurements and lack of apoptotic induction, are consistent with our proposition that CIMSS is membrane impermeable.

### CIMSS inhibits HSV infection and blocks the viral induced phosphorylation of Akt

**Fig. 3:**
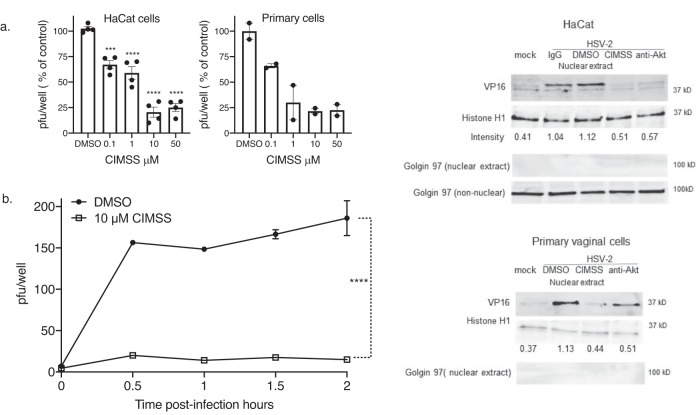
CIMSS inhibits HSV infection. **a** HaCat (*n* = 4) or primary vaginal cells (*n* = 2) were treated with increasing doses of CIMSS (or DMSO control) and then infected with HSV-2(G). Plaques were counted by immunostaining after 48 h and are presented as the percent reduction in viral plaque numbers relative to the DMSO controls, which had ~200 plaques per well (mean ± SD; ***p < 0.001, ***p < 0.0001, ANOVA with multiple comparisons compared to DMSO control for HaCat cells). **b** HaCat cells were synchronously infected with HSV-2(G), treated with media containing 10 μM CIMSS or 0.1% DMSO at temperature shift, and at the indicated times post-temperature shift, extracellular virus was inactived with low pH citrate buffer and cells were overlaid with methylcellulose. Plaques were counted at 48 h (mean ± SEM, *n* = 2). Asterisks indicate significance at each time point by ANOVA (****p < 0.0001). **c** HaCat or primary vaginal epithelial cells were mock or synchronously infected with HSV-2(G) (MOI = 10 pfu/cell) and 0.1% DMSO, 10 μM CIMSS, 2 μg/ml rabbit anti-Akt or a control IgG were added at the time of temperature shift. Nuclear extracts were prepared after 1 h incubation at 37 °C and probed with antibodies for VP16, histoneH1 (nuclear protein) or Golgin-97 (cytoplasmic protein). The blot is representative of results obtained in two independent experiments; intensity of VP16 band (relative to histoneH1) is indicated below each lane.

Given the association of Akt translocation and its phosphorylation with HSV cell entry, we examined whether CIMSS would inhibit HSV infection by plaque assay and found that it inhibited HSV-2 infection of HaCat and primary vaginal epithelial cells in a dose dependent manner with greater than 75% inhibition at a dose of 10 μM (Fig. 3a). Based on these results and the cell viability data, 10 μM CIMSS was used for subsequent studies. The effects of CIMSS or antibodies to Akt on HSV entry were assessed using complementary assays. First, the kinetics of viral entry were compared using a synchronized infection assay. HaCat cells were exposed to 150–200 pfu/well of HSV-2(G) at 4 °C for 4 h to allow virus to bind, washed, transferred to 37 °C (a temperature permissive for viral entry) in fresh media containing 10 μM CIMSS or DMSO. At the indicated times post-temperature shift (0.5, 1, 1.5, or 2 h), the cells were treated with a low pH buffer to inactivate any extracellular virus, washed, overlaid with methylcellulose and viral plaques counted at 48 h. The addition of CIMSS at each timepoint during the viral entry period resulted in a significant reduction in viral plaque formation (Fig. 3b, *p* < 0.001). Second, the effects of CIMSS or anti-Akt antibodies on nuclear transport of the viral tegument protein, VP-16, a surrogate marker for viral entry, were assessed by preparing immunoblots of nuclear extracts 1 h post-infection^11^. The blots were also stained with an antibody to histone 1 (nuclear marker) and anti-golgin-97 (cytoplasmic marker). CIMSS and anti-Akt IgG (but not control IgG) inhibited the nuclear transport of VP-16 in HaCat and primary vaginal cells (Fig. 3c and Supplenetary Fig. 2). The effects of CIMSS or anti-Akt antibodies on viral entry were also assessed by quantifying viral capsid transport to the nuclear pore by confocal imaging^12^. Cells were synchronously infected with HSV-1 K26GFP, which encodes green fluorescent protein fused to VP26^13^, and entry monitored by fixing and staining the cells 1 and 4 h post temperature shift. To differentiate capsid entry from newly synthesized protein, cycloheximide, which blocks protein synthesis, was included as a control. At one hour post-temperature shift, the percentage of GFP + cells was significantly decreased by pretreatment with CIMSS or rabbit-anti-human Akt (*p* < 0.001), but not by cycloheximide. In contrast, the percentage of GFP + cells was significantly reduced by all three treatments 4 h post-temperature shift reflecting inhibition of entry (CMSS and anti-Akt) and inhibition of new protein synthesis (cycloheximide; *p* < 0.0001; Supplementary Fig. 3).

**Fig. 4:**
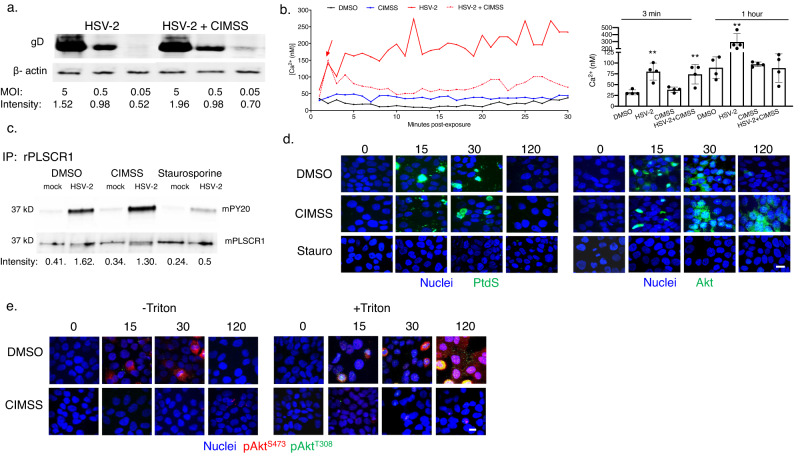
CIMSS inhibits HSV entry downstream of viral binding, activation of phospholipid scramblase, and translocation of phosphatidylserines and Akt to the outer leaflet of the plasma membrane. **a** HaCat cells were exposed to HSV-2(G) at the indicated MOIs in the absence of presence of 10 μM CIMSS for 4 h at 4 °C. The cells were then washed, lysed and Western blots of cell lysates prepared and probed with a mAb to gD as a marker of cell-bound virus and anti-β-actin as a loading control. The blot is representative of results obtained in two independent experiments; relative intensity of gD band after scanning is shown below each lane. **b** HaCat cells were loaded with Fura-2 and then infected with HSV-2(G) (MOI = 10 PFU/cell), or mock-infected in the presence of control buffer (DMSO, 0.1%) or CIMSS (10 µM) and the kinetics of calcium response monitored. Representative responses are shown for the first 30 min (left) and the mean calcium released over the first 3 min and then the extended response in the first hour following viral exposure were calculated from four wells in three independent experiments, each containing 5 × 10^4^ cells (right). Asterisks indicate statistically significant differences by ANOVA relative to DMSO controls (**p < 0.01). **c** HaCaT cells were mock-infected or infected with HSV-2(G) in the presence of 0.1% DMSO, 10 μM CIMSS or 10 μM staurosporine for 30 min and then the cells were lysed and incubated with rabbit anti-PLSCR1 antibody and immune complexes precipitated with protein A-agarose and analyzed by Western blotting with a mouse anti-phosphotyrosine (PY20) or mouse anti-PLSCR mAb. The blot is representative of results obtained in three independent experiments; relative intensity of mPY20 band is shown below. **d** HaCAT cells were infected with HSV-2(G) (MOI = 10 PFU/cell) in the presence of 10 μM CIMSS, 10 μM staurosporine or 0.1% DMSO, and prior to infection (time = 0 min) or after 15, 30, or 120 min, the cells were fixed without permeabilization and stained with mAbs for phosphatidylserines (PtdS) (left panel) or Akt (right panel). Nuclei were stained blue with DAPI. Images were obtained with ZeissLive/DuoScan (objective 100 × 1.4, bar = 10 μm) are representative of two independent experiments. **e** HaCat cells were infected with HSV-2(G) (MOI = 10 PFU/cell) in the presence of 0.1% DMSO or 10 μM CIMSS and at the indicated times, cells were fixed without (-Triton) or with (+Triton) permeabilization and stained with mAbs for phosphorylated Akt (pAkt^S473^, red and pAkt^T308^, green). Nuclei are stained blue (DAPI). Images were obtained with ZeissLive/DuoScan (objective 100 × 1.4, bar = 10 μm) and are representative of two independent experiments; quantification is shown in Supplementary Fig. 5.

CIMSS did not block HSV binding to cells (Fig. 4a and Supplemental Fig. 4) or the initial Ca^2+^ transient, which is detected within the first three minutes following HSV exposure (Fig. 4b). We previously showed that this initial transient triggers phosphorylation (and activation) of PLSCR1^2^. Phosphorylated PLSCR1 was detected by immunoprecipitation (IP) for PLSCR1 and immunoblotting for phosphorylated proteins (there is no antibody specific for phosphorylated PLSCR1). Phosphorylation of PLSCR1 was inhibited by staurosporine, but not by CIMSS, indicating that PLSCR1 is phosphorylated intracellularly (Fig. 4c and Supplementary Fig. 4). Consistent with this interpretation, only staurosporine inhibited the translocation of PtdS and Akt to the outer leaflet of the plasma membrane (Fig. 4d and Supplementary Fig. 5a). PtdS and Akt were detected by microscopy in non-permeabilized cells within 15 min of exposure to HSV, but were no longer detected on the cell exterior at 120 min in DMSO treated cells, in agreement with the previously described kinetics and restoration of the membrane lipid distribution in response to HSV entry^2^. CIMSS did not block the translocation of PtdS and Akt to the outer leaflet, but did reduce levels of phosphorylated Akt (serine 473 and threonine 308) detected in non-permeabilized cells, indicating that these phosphorylation events (unlike PLSCR1 phosphorylation) occur extracellularly (Fig. 4e and Supplementary Fig. 5b). Treatment with CIMSS also resulted in a significant reduction in the extended Ca^2+^ release (quantified for the first hour pi) (Fig. 4b) as well as a decrease in phosphorylated Akt detected in permeabilized cells (Fig. 4e)-findings reflective of our prior obsevations that these cellular responses are triggered by viral entry^2–4^. Notably, Akt (but not PtdS) was still detected in non-permeabilized cells at 120 min in CIMSS but not DMSO treated cells (Fig. 4d). Using a small molecular PLSCR1 inhibitor, we previously demonstrated that PtdS relocalization is a PLSCR1-dependent process^2^. The differences in the repartitioning of PtdS and Akt might reflect different internalization mechanisms. Akt colocalizes with HSV gB in coimmunoprecipitation studies^2^ and could be retained on the outside if viral entry does not occur whereas reinternalization of PtdS may occur independent of viral entry.

### HSV also triggers translocation of PDPK1 and PLCγ and their subsequent phospohorylation is inhibited by CIMSS

**Fig. 5:**
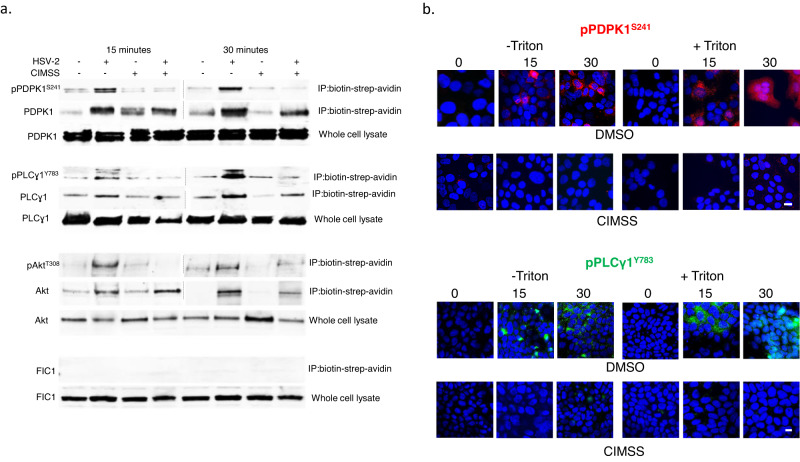
HSV triggers translocation of PDPK1 and PLCγ1 to the outer leaflet and their subsequent phosphorylation is inhibited by CIMSS. **a** HaCat cells were mock-infected or synchronously infected with HSV-2(G) in the absence or presence of 10 µM CIMSS. After 15 min incubation, cell surface proteins were biotinylated and precipitated with streptavidin magnetic beads and analyzed by immunoblotting with Abs to pPDPK1^S241^ and total PDPK1, pPLCγ1^Y783^ and total PLCγ1, pAkt^T308^ and total Akt, and FIC-1 (cytosolic protein); controls include whole cell lysates. Results are representative of two independent experiments. **b** HaCat cells were mock-infected or infected with HSV-2(G) (MOI = 10 PFU/cell) in the presence of 0.1% DMSO or 10 µM CIMSS and at the indicated time post-infection cells were fixed with or without Triton X-100 permeabilization and stained for pPDPK1^S241^ (red, upper panel, ZeissLive/DuoScan, objective 100 × 1.4) or pPLCγ1^Y783^ (green, lower panel, LeicaSP8, objective 63 × 1.4). Representative images from 2–3 independent experiments are shown (bars = 10 μm).

We hypothesized that other kinases typically associated with the inner leaflet might also translocate to the outer leaflet of the plasma membrane in response to PLSCR1 activation and be susceptible to the inhibitory effects of CIMSS. We focused on PDPK1, which activates Akt and other kinases, and PLCγ, which is involved in activating intracellular Ca^2+^ signaling pathways and may be a substrate for phosphorylated Akt^3,14,15^. Prior to HSV exposure, neither PDPK1, PLCγ or Akt were detected in the membrane fraction following biotinylation of cell surface proteins, precipitation with streptavidin beads, and immunoblotting of the precipitated proteins with antibodies for the respective proteins. The proteins were detected in the whole cell lysates. However, within 15 min of HSV exposure, total and phosphorylated PDPK1, PLCγ and Akt were detected in the membrane fraction but their phosphorylation was reduced when the cells were treated with CIMSS (Fig. 5a and Supplementary Fig. 6). As an additional control, blots were probed for FIC-1 (floppase), a cytosolic protein, which was only detected in the whole cell lysates, but not in the membrane fraction. Similar results were obtained by confocal microscopy. No phosphorylated PDPK1 or PLCγ signal was detected in cells prior to HSV exposure, but both phosphorylated proteins were detected in images obtained 15 and 30 min following viral infection and their phosphorylation was inhibited by CIMSS (Fig. 5b).

**Fig. 6:**
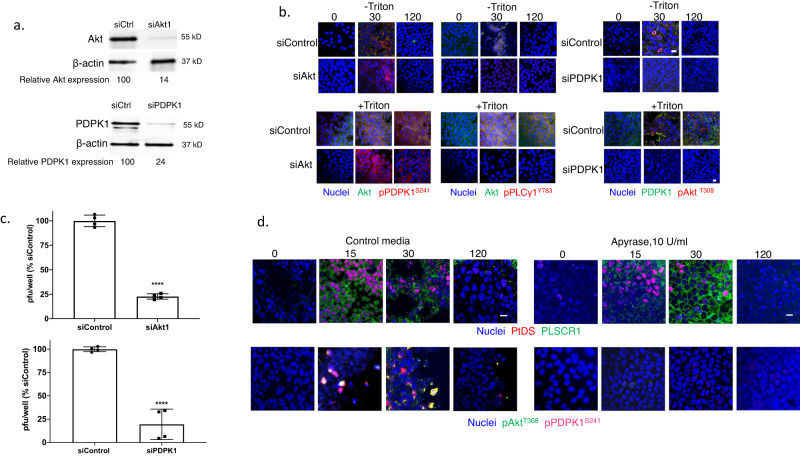
PDPK1 is phosphorylated upstream and PLCγ downstream of Akt phosphorylation at the outer leaflet of the plasma membrane. **a** HaCat cells were transfected with siControl, siAkt1 or siPDPK1 and silencing assessed by preparing Western blots after 72 h and probing for Akt, PDPK1 and β-actin; blots are representative of two independent experiments and relative Akt and PDPK1 expression is quantified after scanning images. **b** The siRNA-transfected cells were exposed to HSV-2(G) (MOI = 10 PFU/cell) (72 h after transfection) and at the indicated times post-infection (0, 30, or 120 min), the cells were fixed and stained (with or without Triton X-100 permeabilization) with antibodies to detect total (green) or phosphorylated Akt^T308^ (red), total (green) or phosphorylated PDPK1^S241^ (red) or pPLCγ1^Y783^ (red); nuclei were stained blue with DAPI. Images are representative of 2 independent experiments (Leica SP8, objective 63 × 1.4, bar = 10 μm). **c** The siRNA-transfected cells were infected with HSV-2(G) (100-200 PFU/well) and plaques were counted at 48 h; results are presented for two independent experiments each performed in duplicate as percent of PFU/well detected in the siControl transfected cells. The asterisks indicate *p* < 0.0001, unpaired *t*-test. **d** HaCat cells were infected with HSV-2(G) (MOI = 10 PFU/cell) in the presence of 10 U/ml of apyrase or control media and at the indicated times post-infection, fixed and stained (without permeabilization) with antibodies to detect phosphatidylserines (PtdS) and phospholipid scramblase (PLSCR1), pAkt^T308^ or pPDPK1^S241^. Images (Leica SP8, objective 63 × 1.4, bar = 10 μm) are representative fields from two independent experiments.

Transfection of HaCat cells with siRNA targeting Akt1 (the dominant isoform) or PDPK1 reduced protein expression as assayed by immunoblots to 14% and 24%, respectively, relative to cells transfected with a control siRNA (Fig. 6a and Supplementary Fig. 7a). The silencing of Akt1 had no effect on HSV-triggered phosphorylation of PDPK1, but resulted in a reduction in phosphorylated PLCγ in non-permeabilized cells. Conversely, silencing of PDPK1 was associated with a reduction in phosphorylated Akt (Fig. 6b). These findings demonstrate that PDPK1 and PLCγ are also translocated to the outer leaflet of the plasma membrane in response to HSV. They further suggest a model, similar to intracellular signaling pathways, in which PDPK1 is activated upstream and PLCγ downstream of Akt phosphorylation when these kinases are translocated to the outer leaflet of the plasma membrane. Silencing of Akt1 and PDPK1 were each associated with a significant reduction in HSV infection by plaque assay (Fig. 6c). To further assess the importance of these extracellular ATP-dependent processes on HSV infection, we conducted studies in the presence of apyrase, a cell-impermeable enzyme that hydrolyzes extracellular ATP to AMP. Apyrase had no discernible effect on the detection of PtdS or PLSCR1 at the outer leaflet by fluorescence microscopy, consistent with intracellular activation of PLSCR1, but inhibited the HSV-induced phosphorylation of Akt and PDPK1 in non-permeabilized cells (Fig. 6d).

### CIMSS blocks viral entry mediated by SARS-CoV-2 Spike protein

**Fig. 7:**
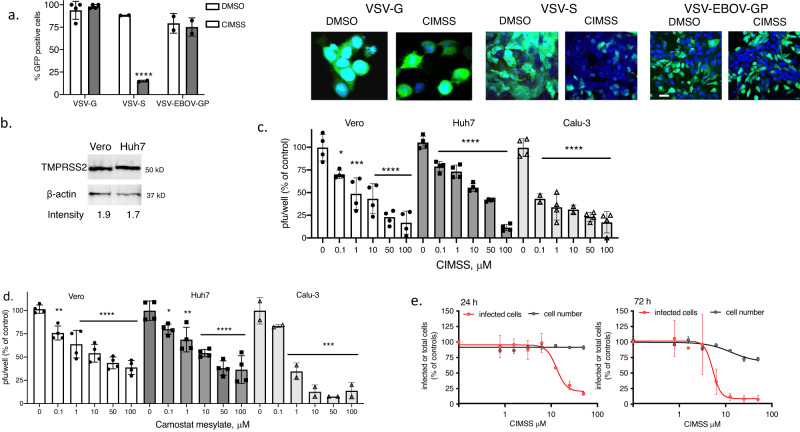
CIMSS inhibits VSV pseudotyped with SARS-CoV-2 spike protein and native SARS-CoV-2 infection. **a** Vero cells were infected with VSV viruses expressing the enhanced green fluorescence protein and VSV glycoprotein G (VSV-G), SARS-CoV-2 spike protein (VSV-S) or Ebola virus glycoprotein (VSV-EBOV-GP) in the presence of 0.1% DMSO or 10 µM CIMSS. The percentage of GFP-positive cells were quantified 24 h after infection by microscopy and results are shown as mean ± SD from two independent experiments with ~200–300 cells counted over 4 fields per experiment (*n* = 4 for VSV-G), ***p < 0.0001 comparing CIMSS versus DMSO for each virus, unpaired *t*-test. Representative images are shown on the right, images with VSV-G were obtained with ZeissLive/DuoScan (objective 100 × 1.4) and for VSV-S and VSV-EBOV-GP with Leica SP8 (objective 63 × 1.4) (bar = 10 μm). **b** Western blots of Vero or Huh7 cell lysates probed for TMPRSS2 and β-actin as loading control. The intensity of TMPRSS2 relative to β-actin is indicated below each lane; blots are representative of two independent experiments. Vero, Huh7 or Calu-3 cells were infected with the VSV-S in the presence of 0.5% DMSO or increasing concentrations of CIMSS (**c**) or camostat mesylate (**d**) and plaques counted after 48 h infection or, for Calu-3 cells, culture supernatants were harvested and infectious viral yields quantified by titering on Vero cells. Asterisks indicate significance relative to the DMSO control for each virus (ANOVA, *p < 0.05, **p < 0.01, ***p < 0.001 and ****p < 0.0001). Results are mean ± SD from 2 to 4 independent experiments conducted in duplicate. **e** Huh-7.5 cells were treated with CIMSS 1 h prior to infection with SARS-CoV-2 (strain: WA1/2020). Infections were performed at MOIs of 0.25 PFU/cell for a 24 h timepoint and 0.01 PFU/cell for a 72 h timepoint. Cells were subsequently stained for the nucleocapsid protein; nuclei were stained using Hoechst 33342. The percentage of SARS-CoV-2 positive cells and nuclei (cell number) are presented relative to the DMSO control. Error bars represent SEM for *n* = 3 replicates.

In addition to providing a tool to identify proteins that undergo phosphorylation at the outer leaflet of the plasma membrane, susceptibility to CIMSS may identify other viruses that exploit PLSCR1-dependent signaling pathways to promote viral entry. To test this hypothesis, we evaluated the inhibitory effects of CIMSS on vesicular stomatitis virus (VSV) expressing its native glycoprotein G (VSV-G), the Ebola virus glycoprotein (VSV-EBOV-GP) or SARS-CoV-2 spike protein (VSV-S); each of these also express enhanced GFP for tracking^16,17^. VSV and EBOV enter cells by endocytosis^18,19^, whereas SARS-CoV-2 enters by fusion of the viral envelope with the cell plasma membrane as well as by endocytosis^20,21^. CIMSS (10 μM) had no effect on VSV-G or VSV-EBOV-GP, but significantly reduced VSV-S infection of Vero cells as monitored by quantifying the percentage of GFP^+^ cells (Fig. 7a). We extended the studies with VSV-S to include the human cell lines, Huh7 and Calu-3. SARS-CoV-2 infection of Calu-3 cells has been shown to be highly dependent on expression of the cellular protease TMPRSS2, which triggers the cleavage of spike to release the S2 fusion subunit and is insensitive to chloroquine, an inhibitor of endocytosis^20,21^. Western blots demonstrated that Huh7 and Vero cells also express TMPRSS2 (Fig. 7b and Supplementary Fig. 7b). Both CIMSS and camostat mesylate, a protease inhibitor that blocks TMPRSS2 activity, indvidually inhibited VSV-S infection of all 3 cell lines (Calu-3, Vero and Huh7) in a dose dependent manner (Fig. 7c, d). Importantly, CIMSS exhibited inhibitory activity against authentic SARS-CoV-2 (WA1/2020) infection of human Huh-7.5 cells at 24 and 72 h. The percentage of infected cells was determined by automated microscopy after staining for viral nucleoprotein to identify infected cells and for total cell number by nuclear staining with Hoechst 33342 (Fig. 7e).

**Fig. 8:**
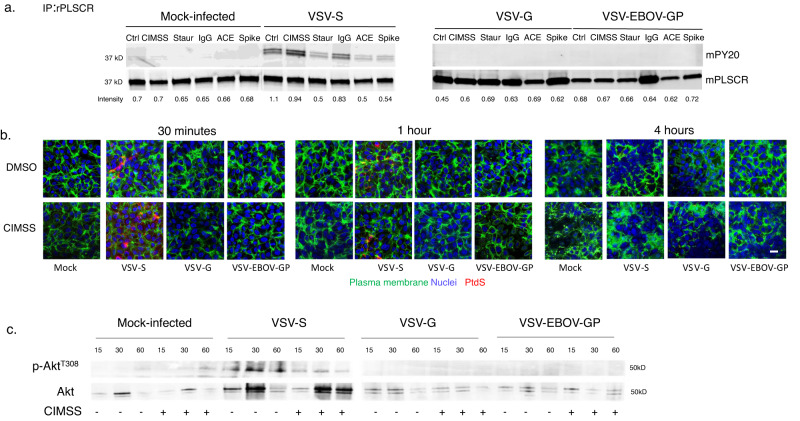
VSV-S triggers activation of phospholipid scramblase and the translocation of phosphatidylserines. **a** Vero cells were mock-infected or infected with the indicated viruses for 30 min in the absence or presence of CIMSS or staurosporine (10 μM each) or murine anti-ACE2 (anti-ACE), anti-Spike (anti-S) or an isotype control IgG (10 μg/ml of each immunoglobulin). The cells were lysed and incubated with rabbit anti-PLSCR1 antibody and immune complexes precipitated with protein A-agarose and analyzed by western blotting with a mouse anti-phosphotyrosine (PY20) or mouse anti-PLSCR mAb. The blot is representative of results obtained in two independent experiments. **b** Vero cells were stained for plasma membranes (green) with wheat germ agglutinin conjugated with Alexa488 and then mock-infected or infected with the indicated viruses in the presence of 0.1% DMSO or 10 μM CIMSS and after 30 min, 1 h, or 4 h, fixed and stained with an antibody to phosphatidylserines (PtdS) (red); nuclei were stained with DAPI. Images were obtained with Leica SP8 (objective 63 × 1.4, bar = 10 μm). **c** Vero cells were mock-infected or infected with the indicated VSV pseudotyped viruses in the absence or presence of 10 µM CIMSS. After 15, 30, or 60 min, the cell surface proteins were biotinylated and precipitated with streptavidin magnetic beads and analyzed by immunoblotting with Abs to pAkt^T308^ and total extracellular Akt. Results are representative of two independent experiments.

To directly address the role of the PLSCR1 on entry mediated by the SARS-CoV-2 spike protein, we assessed phosphorylation of immunoprecipitated PLSCR1 following exposure of Vero cells to the VSV pseudotyped viruses. As observed with HSV, VSV-S, but not VSV-G or VSV-EBOV-GP triggered phosphorylation of PLSCR1. Phosphorylation of PLSCR1 was preserved in the presence of CIMSS, but was inhibited by staurosporine, as well as by treatment with anti-ACE2 or anti-Spike antibodies (Fig. 8a and Supplementary Fig. 8). VSV-S triggered PLSCR1 phosphorylation was associated with translocation of PtdS to the outer leaflet as assessed by confocal microscopy, which peaked at 30 min with restoration of lipid distribution by 4 h (Fig. 8b and Supplementary Fig. 5c). Moreover, VSV-S (but not VSV-G or VSV-EBOV-GP) triggered extracelluar Akt translocation and phosphorylation as assessed following biotinylation of cell surface proteins, precipitation with streptavidin beads, and immunoblotting for pAkt^T308^ or total Akt; the extracellular phosphorylation of Akt was inhibited by CIMSS (Fig. 8c and Supplementary Fig. 9). Similar results were obtained by confocal microscopy; Akt and pAkt as well as PDPK1 and pPDPK1 were detected in non-permeablized cells in response to VSV-S and their phosphorylation was inhibited by CIMSS (Supplementary Fig. 10).

**Fig. 9:**
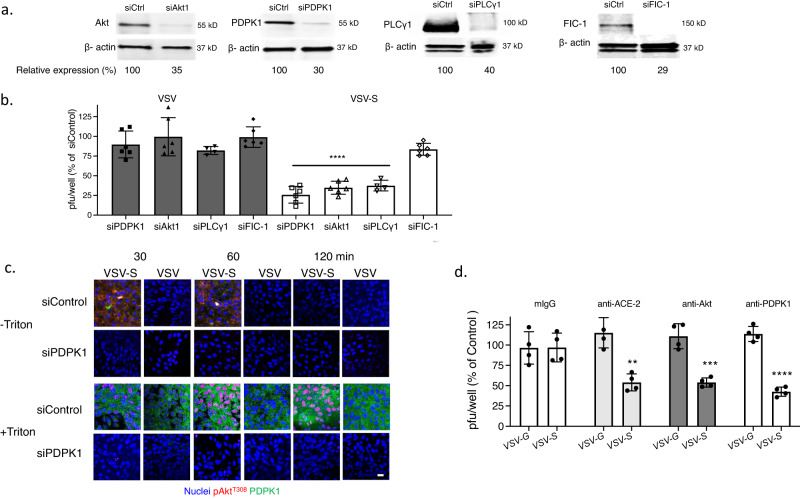
Silencing of PDPK1, PLCγ1, and Akt inhibits VSV-S infection. **a** Vero cells were transfected with control siRNA or siRNA targeting Akt1, PDPK1, PLCγ, or FIC-1 and after 72 h, cell lysates were assayed by preparing western blots and probing for respective proteins. Cell lysates were probed for β-actin on separate blots. Blots were scanned and and the percent reduction in protein expression of the silenced protein relative to siControl-transfected cells is indicated; blots are representative of two independent experiments. **b** Silenced cells were infected in duplicate with VSV-G or VSV-S and plaques quantified after 48 h incubation. Results are presented as mean ± SD and asterisks indicate significance relative to plaques formed on siControl wells (ANOVA, ****p < 0.0001). **c** Vero cells were transfected with siPDPK1 or control siRNA as in **a** and then infected with VSV-G or VSV-S. Following incubation for 30, 60, or 120 min, cells were fixed without or with Triton X-100 permeabilization and stained with antibodies to pAkt^T308^ (red) or PDPK1 (green); nuclei were stained with DAPI. Images (Leica SP8, objective 63 × 1.4) are representative of results obtained in two independent experiments (bar = 10 μm). **d** Vero cells were infected with VSV-S or VSV-G in the presence of antibodies that target Akt (rabbit polyclonal), PDPK1 (rabbit polyclonal), the ACE2 receptor (murine monoclonal), or an murine monoclonal isotype control for 1 h, washed, and infection monitored by counting plaques 24 h pi. Results are presented as mean ± SD and asterisks indicate significance relative to plaques formed on isotype control antibody treated wells (**p < 0.01, ***p < 0.001, ****p < 0001).

To evaluate whether this pathway contributed to VSV-S entry, Vero cells were transfected with siRNA targeting Akt1, PDPK1, PLCγ1, or FIC-1 (negative control). Targeted protein expression was reduced to 29–40% of expression detected in cells transfected with control siRNA, as judged by western blot (Fig. 9a and Supplementary Fig. 11). Silencing of PDPK1, Akt1 and PLCγ1 resulted in significant reduction of VSV-S but not VSV-G infection (Fig. 9b). Furthermore, silencing of PDPK1 prevented VSV-S induced phosphorylation of Akt following VSV-S infection as evidenced by microscopy of non-permeabilized and permeabilized cells (Fig. 9c). To further assess the role of this signaling pathway in VSV-S entry, Vero cells were infected with virus in the presence of antibodies that target Akt, PDPK1, the human ACE2 receptor, or an isotype control for 1 h, washed, and infection monitored by counting plaques 24 h pi. Anti-ACE2 (murine mAb), anti-Akt (rabbit polyclonal), and anti-PDPK1 (rabbit polyclonal) significantly inhibited VSV-S, but not VSV-G infection (Fig. 9d).

## Discussion

We synthesized a cell impermeable analog of staurosproine, CIMSS, which represents the prototype of a new class of tool compounds (to the best of our knowledge). Impermeability of this analog was demonstrated by its partitioning properties, lack of cytotoxicity, inability to induce apoptosis, and failure to inhibit intracellular Akt phosphorylation in response to insulin at concentrations 100–1000-fold greater than the parental drug. The higher concentrations of CIMSS were used in these assays because the analog is generaly not as potent and exhibits reduced IC_50_s relative to staurosporine for a range of kinases. This behavior is not unexpected, as the amide formed by acylation possesses altered hydrogen bonding capabilities relative to the initial secondary amine, and in some cases may impose unfavorable sterics^10^. This argument has been invoked to explain why a stauropsorine derivative acylated at the 4′-methylamine exhibits an ~80-fold reduction in affinity for ASK1/MAP3K5 relative to staurosporine^22^, although there is not complete concordance in the literature as a different acylated staurospine analog exhited an IC_50_ for PKA very similar to staurosporine^23^.

Importantly, CIMSS retains the broad inhibitory profile characteristic of staurosporine. This promiscuity, coupled with impermeability, makes CIMSS an excellent tool compound for examing unique kinase-dependent processes that are occurring at the outer leaflet of the plasma membrane or extracellularly. CIMSS enabled the identification of other cellular proteins (PDPK1 and PLCγ) that are translocated to the extracellular milieu following activation of PLSCR1 by HSV triggered calcium transients. Moreover, CIMSS also uncovered the ability of SARS-CoV-2 spike protein to activate this outside-in PLSCR1-Akt signaling pathway. This process is distinct from the previously described intracellular activaton of the phosphatidylinositol 3-kinase/Akt signaling pathway, which among other intracellular processes, regulates clathrin-mediated endocytosis of SARS-CoV-2 and other viruses^24,25^. However, the intracellular activation of Akt signaling would not be susceptible to CIMSS as illustrated by the studies with insulin. Notably, HIV also triggers the externalization of PtdS to promote membrane fusion through the activation of a different phospholipid scramblase, TMEM16F, but whether this is associated with externalization of cellular kinases and thus whether HIV entry would be inhibited by CIMSS is not yet known^26^.

Specifically, using CIMSS to discriminate whether a phosphorylation event occurs intracellularly or in association with the outer leaflet of the plasma membrane, we demonstrated that in response to canonical activation of the insulin receptor, Akt is phosphorylated only intracellularly, whereas HSV and VSV-S infection also induce extracellular Akt phosphorylation. In contrast, PLSCR1 itself was phosphorylated intracellularly in response to virally induced Ca^2+^ transients as evidenced by its inhibition by unmodified staurosporine but its insensitivity to CIMSS. Focusing on proteins associated with intracellular Akt signaling, we demonstrated that PLSCR1 activation also resulted in the translocation of PDPK1 and PLCγ to the outer leaflet of the plasma membrane where they are subsequently phosphorylated, as evidenced by confocal imaging, biotinylation of cell membranes, and susceptibility to blockade of these processes by CIMSS. By analogy with the canonical cytoplasmic signaling pathways^3,14,15^, we propose that PDPK1 participates in extracellular autophosphorylation and that the resulting activated phosphorylated PDPK1 is likely responsible for the subsequent phosphorylation of outer leaflet Akt since silencing of PDPK1 prevented outer leaflet plasma membrane Akt phosphorylation. Furthermore, activated phosphorylated Akt likely elicits PLCγ phosphorylation, as silencing of Akt results in a reduction in PLCγ phosphorylation at the outer leaflet of the plasma membrane. Notably, CIMSS inhibited PDPK1, Akt, and PLCγ phosphorylation and reduced HSV entry and infection, highlighting the importance of extracellular kinase function/phosphorylation events in viral infection. Evidence for a role of extracellular kinase activity in HSV entry is further provided by the studies with apyrase, which inhibited the phosphorylation of Akt and PDPK1, and with our studies showing that antibodies targeting Akt also inhibit HSV entry and subsequent infection^2^.

CIMSS also served as an effective tool to identify other viruses that might exploit a similar kinase-dependent signaling pathway to promote viral entry. Specifically, we found that VSV pseudotyped with SARS-CoV-2 spike, but not viruses expressing the native VSV glycoprotein G or EBOV glycoprotein, were susceptible to inhibition by CIMSS. Notably, authentic SARS-CoV-2 was similarly inhibited as VSV-S. As observed with HSV, VSV-S activated phospholipid scramblase to promote translocation of PtdS, PDPK1, and Akt to the outer leaflet and the latter two kinases were subsequently phosphorylated in a CIMSS-sensitive manner. Silencing of PDPK1, Akt1, or PLCγ resulted in a reduction in VSV-S infection supporting the importance of this signaling pathway. Additional support for the role of extracellular kinases in VSV-S comes from observation that polyclonal or monoclonal Abs targeting Akt or PDPK1 inhibited VSV-S infection. While the antibody studies do not distinguish between mechansims involving reductions in catalytic activity or steric blockade of protein-protein interactions involving these kinases, these results, combined with the observation that CIMSS inhibits VSV-S, provide strong evidence that both Akt and PDPK1 extracellular signaling are directly involved in HSV and VSV-S (and native SARS-CoV-2) entry.

VSV and EBOV primarily enter cells via endocytosis and their entry is effectively blocked by inhibitors of this pathway^18,19^. For example, EBOV enters by macropinocytosis and traffics through the endosomal pathway where cathepsin-dependent cleavage of EBOV-GP occurs. Subsequently, the cleaved viral glycoprotein interacts with Niemann-Pick C1 (NPC1), a late endosome/lysosome resident host protein, to trigger viral and intracellular membrane fusion, resulting in release of the viral genome. While this intracellular fusion step is blocked by inhibitors of receptor tyrosine kinases, which interfere with post-internalization signaling events^27^, we demonstrated this process was insensitive to CIMSS, consistent with the impermeability and extracellular activity of CIMSS. In contrast, HSV-1 and HSV-2 primarily enter human keratinocytes and other epithelial cells by direct fusion between the viral envelope and the plasma membrane, which generates a lipid pore that permits the viral genome-containing capsid (and associated tegument proteins) to be released intracellularly^28^. This plasma membrane-viral envelope fusion event is blocked by inhibitors of the PLSCR1-Akt signaling pathway^2–5^, including, as shown here, the cell impermeable inhibitor of Akt phosphorylation, CIMSS, as well as anti-Akt and anti-PDPK1 antibodies. The observation that SARS-CoV-2 is also inhibited by CIMSS and that VSV-S activates a PLSCR1-Akt signaling pathway similar to that observed for HSV supports the notion that SARS-CoV-2 enters these cells, at least in part, by direct fusion. Although there has been controversy about the relative role of fusion of the viral envelope with plasma membrane versus endocytosis for entry of SARS-CoV-2 into different cell types, our studies support a role for direct fusion pathway for viral entry in the cells studied here. We found that all three cell types studied (Vero, Huh7, and Calu-3 cells) were susceptible to inhibition by both CIMSS and camostat mesylate, an inhibitor of TMPRSS2. These results are consistent with studies showing that camostat mesylate is more effective than ammonium chloride, which increases endosomal pH to block cathepsin activity, or other inhibitors of endocytosis at inhibiting SARS-CoV-2 infection^20,21^. It should be noted that CIMSS did not abolish VSV-S or HSV infection, which may reflect the ability of both viruses to use alternative endocytic pathways for entry.

**Fig. 10:**
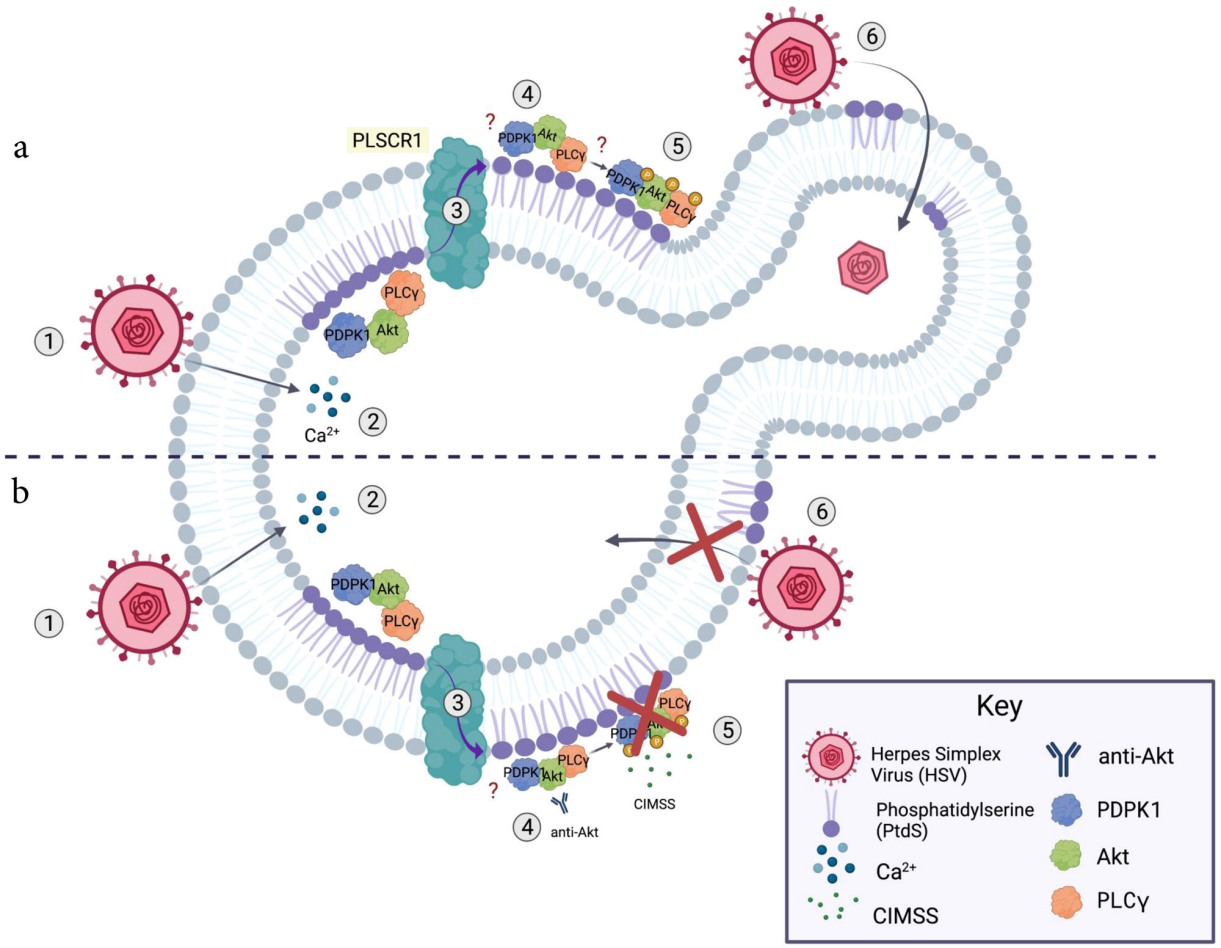
Model of how cell-impermeable kinase inhibitors block viral entry. **a** Binding of HSV to cellular receptors (1) triggers intracellular calcium transients (2), which activate phospholipid scramblase-1 (3) leading to the translocation of phospatidylserines and cellular proteins (PDPK1, Akt, PLCγ and possibly others) to the outer leaflet of the plasma membrane (4) where, analogous to the the canonical cytoplasmic signaling pathways, autophosphorylation of PDPK1 triggers phosphorylation of outer leaflet Akt, which, in turn, phosphorylates PLCγ (5). This signaling pathway is required for HSV entry and is associated with subsequent restoration of phospholipid distribution (6). **b** Cell-impermeable kinase inhibitors (e.g. CIMSS) or antibodies to Akt block the activation of this extracellular signaling pathway (5) and prevent HSV entry resulting in (6) persistence of extracellular PtdS, which may lead to apoptosis.

In summary, we have exploited the unique properties of CIMSS to examine viral entry mechanisms of HSV and SARS-CoV-2, and to identify unique extracellular interactions and catalytic contributions to these processes (Fig. 10). Future efforts to define the contribution of extracellular kinase activities to viral entry will benefit from more selective reagents, including mechanistically defined antibodies and impermeable analogs of selective kinase inhibitors. While we focused on CIMSS as a tool compound, our results suggest that cell impermeable kinase inhibitors may also represent a novel (to the best of our knowledge) candidate class of antiviral drugs. Advantages include their safety profile because of their cell impermeability, which reduces off-target engagement, as interactions with cytoplasmic proteins are precluded, and the likelihood that this class of inhibitors is less prone to select for viral escape mutants because they target host proteins and not the virus. The previously unappreciated translocation of kinases to the extracellular milieu and the subsequent activation/phosphorylation of these kinases at the outer leaflet may have implications for other biological processes associated with calcium transients that activate phosphoplipid scramblase.

## Methods

### Synthesis of CIMSS

All chemical reagents and solvents were obtained from commercial sources and used without further purification. Microwave reactions were performed using an Anton Paar Monowave 300 reactor. Chromatography was performed on a Teledyne ISCO CombiFlash Rf 200i using disposable silica cartridges. Analytical thin layer chromatography (TLC) was performed on Merck silica gel plates and compounds were visualized using UV or CAM stain. NMR spectra were recorded on a Bruker 600 spectrometer. 1H chemical shifts (δ) are reported relative to tetramethyl silane (TMS, 0.00 ppm) as internal standard or relative to residual solvent signals.

### Step 1

**Tert-Butyl ((1-(3-hydroxypropyl)-1*H*-1,2,3-triazol-4-yl)methyl)carbamate (1)**: *N*-Boc-propargylamine (3.1 g, 20 mmol), 3-azidopropan-1-ol (2.0 g, 20 mmol) and THF (50 mL) were combined in a flask which was then purged with Ar. (PPh_3_)_3_CuBr (150 mg, 0.16 mmol, 0.8 mol%)^29^ was added and the resulting mixture was stirred at room temperature for 5 days. EDTA-Na_4_ (300 mg) was added and the volaties were removed under reduced pressure. The crude product was loaded on a silica cartridge with CH_2_Cl_2_ and then purifed by flash chromatography (24 g silica, 0–25% Ultra [CH_2_Cl_2_:MeOH:NH_4_OH 75:23:2] in DCM). Minor impurities were removed by a second chromatographic separation (0-100% acetone in hexanes) to give the pure product as a clear oil (4.9 g, 20 mmol, 97%).

**TLC***: R*_f_ = 0.31 (CH_2_Cl_2_:Ultra 3:1; CAM). ^**1**^**H NMR (600** **MHz, CHCl**_**3**_**)** δ 7.57 (s, 1H), 5.21 (bs, 1H), 4.51 (t, *J* 6.8 Hz, 2H), 4.38 (d, *J* 6.0 Hz, 2H), 3.64 (q, *J* 5.5 Hz, 2H), 2.34 (t, *J* 5.1 Hz, 1H), 2.12 (p, *J* 6.3 Hz, 2H), 1.44 (s, 9H). ^**13**^**C NMR (151** **MHz, CDCl**_**3**_**)** δ 155.93, 145.43, 122.43, 79.77, 58.67, 46.92, 36.08, 32.56, 28.39. **ESI-MS:** calc’d for C_11_H_20_N_4_O_3_ (M + H)^+^ 257.1608 found 257.1602.

### Step 2

**Tert-Butyl ((1-(3-bromopropyl)-1*H*-1,2,3-triazol-4-yl)methyl)carbamate (2)**: a flask containing alcohol **1** (2.90 g, 11.3 mmol, 1.0 equiv.) was purged with argon and closed with a septum/Ar balloon. THF (100 mL) was added followed by PPh_3_ (5.94 g, 22.6 mmol, 2.0 equiv.) and CBr_4_ (7.50 g, 22.6 mmol, 2.0 equiv.). A precipitate formed within 15 min. The resulting mixture was stirred at room temperature for 17 h, before being diluted with Et_2_O (50 mL) and filtered. The solids were rinsed with Et_2_O (50 mL). Volatiles were removed and the residue purified by column chromatography (24 g silica, 0–40% acetone in hexanes). The product was obtained as an oil that crystalized upon standing (2.53 g, 7.93 mmol, 70%).

**TLC**: *R*_f_ = 0.29 (hexanes:acetone 1:1; I_2_ then CAM). ^**1**^**H NMR** **(600** **MHz, CDCl**_**3**_**)** δ 7.57 (s, 1H), 5.11 (bs, 1H), 4.53 (t, *J* 6.6 Hz, 2H), 4.40 (d, *J* 6.0 Hz, 2H), 3.36 (t, *J* 6.2 Hz, 2H), 2.46 (p, *J* 6.4 Hz, 2H), 1.44 (s, 9H). ^**13**^**C NMR (151** **MHz, CDCl**_**3**_**)** δ 155.85, 145.53, 122.49, 79.75, 48.10, 36.09, 32.55, 29.32, 28.37. **ESI-MS:** calc’d for C_11_H_20_BrN_4_O_2_ (M + H)^+^ 319.0764 found 319.0766.

### Step 3

**Sodium 3-(4-(((tert-butoxycarbonyl)amino)methyl)-1*H*-1,2,3-triazol-1-yl)propane-1-sulfonate (3)**: Bromide **2** (530 mg, 1.66 mmol, 1.0 equiv.), ethanol (2 mL), water (1 mL) and sodium sulfite (523 mg, 4.15 mmol, 2.5 equiv.) were added to a microwave vial. The vial was capped and heated to 80 °C for 6 h. Mass spectrometric analysis showed full conversion of the starting material. The insoluble material was removed by filtration (glasswool) and rinsed with ethanol. The liquid phase was concentrated almost to dryness and the product was precipitated by addition of acetone (5 mL). The solids were collected by filtration and rinsed with acetone and CH_2_Cl_2_ to give the sulfonate salt in good purity (310 mg, 0.91 mmol, 55%).

^**1**^**H NMR (600** **MHz, D**_**2**_**O)** δ 7.92 (s, 1H), 4.57 (t, *J* 6.8 Hz, 2H), 4.34 (s, 2H), 2.87 (t, *J* 7.7 Hz, 2H), 2.34 (p, *J* 7.0 Hz, 2H), 1.43 (s, 9H). ^13^C NMR (151 MHz, D_2_O) δ 181.36, 157.97, 123.52, 81.36, 48.72, 47.61, 30.21, 27.57, 25.15. **ESI-MS:** calc’d for C_11_H_20_N_4_O_5_Na (M + H)^+^ 343.1047 found 343.1044.

### Step 4

**Sodium 3-(4-(aminomethyl)-1*H*-1,2,3-triazol-1-yl)propane-1-sulfonate (4)**: Boc-protected amine **4** (53 mg, 0.15 mmol) was dissolved in water and heated to 150 °C for 30 min in a microwave vial. The product was obtained as a white powder after freeze-drying (38 mg, 0.15 mmol, >99%).

^**1**^**H NMR (600** **MHz, D**_**2**_**O)** δ 8.02 (s, 1H), 4.60 (t, *J* 6.9 Hz, 2H), 4. 08 (s, 2H), 2.93–2.80 (m, 2H), 2.36 (p, *J* 7.0 Hz, 2H). ^**13**^**C NMR (151** **MHz, D**_**2**_**O)** δ 145.38, 123.99, 48.74, 47.58, 34.99, 25.11. **ESI-MS:** calc’d for C_6_H_12_N_4_O_3_S (M + H)^+^ 243.0523 found 243.0524.

### Step 5

**Staurosporine** ***N*-4-oxobunanoic acid (5)**^23,30^; Staurosporine (16 mg, 34 mmol, 1.0 equiv.), succinic anhydride (10 mg, 0.10 mmol, 2.9 equiv.), DMAP (8.4 mg, 69 mmol, 2.0 equiv.) and DMSO (1 mL) were combined in a vial, and the resulting solution was stirred overnight while being protected from light. The reaction mixture was diluted with EtOAc (5 mL) and transferred to a Falcon tube containing water (5 mL) and 1 M HCl (1 mL). The phases were separated and the aqueous layer was extracted with EtOAc (3 × 2 mL). The combined organic layers were combined, dried (Na_2_SO_4_), filtered, and concentrated. The residues was loaded on a silica cartridge with CH_2_Cl_2_ and a minimum of MeOH. The product was obtained as an off-white solid after chromatography (4 g silica, 0–10% MeOH in CH_2_Cl_2_). Yield: 18 mg, 32 mmol, 93%.

**TLC**: *R*_f_ = 0.17 (CH_2_Cl_2_:MeOH 9:1; UV). ^**1**^**H NMR (600** **MHz, DMSO-*d6*)** δ 9.29 (d, *J* = 7.8 Hz, 1H), 8.59 (s, 1H), 8.15 (d, *J* = 6.3 Hz, 2H), 8.06 (d, *J* = 7.8 Hz, 1H), 7.99 (d, *J* = 8.4 Hz, 1H), 7.67 (d, *J* = 8.2 Hz, 1H), 7.53–7.46 (m, 2H), 7.36 (t, *J* = 7.5 Hz, 1H), 7.30 (t, *J* = 7.5 Hz, 1H), 7.03 (dd, *J* = 8.5, 6.7 Hz, 1H), 6.77 (d, *J* = 7.1 Hz, 2H), 5.00 (s, 3H), 2.82 (s, 3H), 2.77 (s, 3H), 2.68 (d, *J* = 10.9 Hz, 1H), 2.62–2.56 (m, 1H), 2.24 (td, *J* = 13.1, 6.7 Hz, 1H).

### Step 6

**NaCIMSS:** Acid **5** (18 mg, 32 mmol, 1.0 equiv.) and amine **4** (15 mg, 65 mmol, 2.0 equiv.) were disolved in DMSO (1 mL). Hünig’s base (28 mL, 0.16 mmol, 5.0 equiv.), DMAP (4 mg, 32 mmol, 1.0 equiv.) and EDC (12 mg, 64 mmol, 2.0 equiv.) were added and the mixture was stirred at room temperature overnight while being protected from light. TLC (Ultra^2^, UV) showed practically complete conversion and the solvent was removed under high vacuum. The crude product was dissolved in methanol and absorbed on silica. NaCIMSS was obtained as a white powder after column chromatograpy (4 g silica, Pump A: EtOAc:acetone 4:1. Pump B: MeOH:H_2_O 1:1; 0-20% B). 3.0 mg, 12%.

**TLC**: *R*_f_ = 0.45 (EtOAc:Acetone:MeOH:H_2_O 4:1:1:1; UV). ^**1**^**H NMR (600** **MHz, DMSO-*d6*)** δ 9.29 (d, *J* = 7.8 Hz, 1H), 8.60 (s, 1H), 8.37 (t, *J* = 5.6 Hz, 1H), 8.06 (d, *J* = 7.9 Hz, 1H), 8.01 (d, *J* = 8.5 Hz, 1H), 7.95 (s, 1H), 7.69 (d, *J* = 8.2 Hz, 1H), 7.49 (td, *J* = 7.4, 3.5 Hz, 2H), 7.36 (t, *J* = 7.5 Hz, 1H), 7.30 (t, *J* = 7.4 Hz, 1H), 7.06 (dd, *J* = 8.4, 6.9 Hz, 1H), 5.01 (s, 3H), 4.46 (t, *J* = 7.0 Hz, 3H), 4.32 (d, *J* = 5.6 Hz, 2H), 4.24 (s, 1H), 2.83 (s, 3H), 2.78 (s, 3H), 2.72–2.58 (m, 4H), 2.47–2.37 (m, 5H), 2.34 (s, 3H), 2.22 (td, *J* = 13.0, 6.7 Hz, 1H), 2.13–2.06 (m, 2H). ^**13**^**C NMR (151** **MHz, DMSO*-d6*)** δ 172.62, 172.36, 171.81, 145.36, 139.36, 136.73, 133.12, 126.11, 125.78, 125.46, 124.20, 123.37, 123.07, 121.88, 120.74, 119.89, 119.83, 115.65, 114.56, 114.17, 109.51, 95.13, 83.70, 82.72, 60.96, 55.39, 48.97, 48.57, 48.51, 45.88, 34.79, 31.28, 30.58, 29.96, 29.05, 27.24, 27.07, 27.05. **ESI-MS:** calc’d for C_38_H_40_N_8_O_8_S (M + H)^+^ 791.2582 found 791.2513.

### In vitro kinase assays studies

Kinase activity were performed by Reaction Biology Corporation using the “HotSpot” assay platform modified from the published procedure^31^. Briefly, into a base reaction buffer (20 mM Hepes (pH 7.5), 10 mM MgCl_2_, 1 mM EGTA, 0.01% Brij35, 0.02 mg/mL BSA, 0.1 mM Na_3_VO_4_, 2 mM DTT, 1% DMSO) containing substrate were delivered sequentially (1) required cofactors, (2) kinase enzyme (followed by gentle mixing), (3) compound (CIMSS or controls) in 100% DMSO (introduced by acoustic technology (Echo550; nanoliter range, followed by incubation at 20 °C for 20 min), and finally (4) ^33^P-ATP to initiate the reaction. After two hours, kinase activity was detected by the P81 filter-binding method. The concentration of drug that inhibited 50% of kinase activity (IC_50_) was calculated relative to controls.

### Cell permeability

MDCK Transport analysis was performed by Quintara Discovery (Hayward, CA). MDCK-MDR1 cell plates were maintained for 3 days at 37 °C with 5% CO_2_. Cells were washed with Hank’s Balanced Salt Solution (HBSS) with 5 mM HEPES for 30 min before starting the experiment. Test compound (CIMSS and controls digoxin and propranolol) solutions were prepared by diluting from DMSO stock into HBSS buffer to a final concentration of 5 μM. Prior to the experiment, cell monolayer integrity was verified by transendothelial electrical resistance (TEER). All of the wells had high resistance above the acceptance cut-off (1 kΩ). The test compounds were added to the apical (75 μL) side with blank buffer on the basal side (250 μL). A P-glycoprotein (gp) inhibitor (GF120918, 10 μM) was maintained in the transport buffer to block the active P-gp transporter. Transport plates were incubated at 37 °C in a humidified incubator with 5% CO_2_. A samples was obtained from the donor compartment at time zero and from donor and acceptor compartments after 1 h and analyzed by liquid chromatography with tandem mass spectrometry (LC/MS/MS). Apparent permeability (Papp) values were calculated using the equation: Papp = (dQ/dt)/A/C0 where dQ/dt is the initial rate of amount of test compound transported across cell monolayer, *A* is the surface area of the filter membrane, and C0 is the concentration of the test compound at time zero. All samples were analyzed on LC/MS/MS using an AB Sciex API 4000 instrument, coupled to a Shimadzu LC-20AD LC Pump system. Analytical samples were separated using a Waters Atlantis T3 dC18 reverse phase HPLC column (20 mm × 2.1 mm) at a flow rate of 0.5 mL/min. The mobile phase consisted of 0.1% formic acid in water (solvent A) and 0.1% formic acid in 100% acetonitrile (solvent B). Elution conditions are detailed below:

LC/MS/MS gradient conditions

**Table:** 

Time (min)	Flow (μL/min)	%A	%B
0	500	98	2
0.30	500	98	2
1.40	500	2	98
2.00	500	2	98
2.01	500	98	2
2.50	500	98	2

PAMPA analysis was performed by Quintara Discovery (Hayward, CA). Pre-coated 96-well PAMPA plate system was purchased from Corning and left to thaw to the room temperature for at least 30 min before use. Compounds (CIMSS and controls: atenolol and propranolol) were dissolved at 100 μM in PBS, pH 7.4 with a final DMSO concentration of 1%. The transport experiment was initiated by adding test compounds to the donor (300 μL) side and with blank buffer on the receiver side (200 μL) side. The transport plate was incubated at the room temperature for 5 h. Sampels were obtained from the donor compartment at time zero and from the donor and acceptor compartments after 5 h and analyzed by liquid chromatography with tandem mass spectrometry (LC/MS/MS).

### Cells and viruses

Vero (monkey kidney epithelial cells), HaCat (human keratinocyte spontaneously immortalized cells), Caco-2 cells (*H. sapiens*, sex: male, colon epithelial) (obtained from the American Type Culture Collection (ATCC), Manassas, VA), Calu-3 cells (human lung adenocarcinoma epithelial cells, ATCC), Huh7 (provided by K. Chandran) and the derivative Huh-7.5 hepatoma cells^32^ were cultured in Dulbecco’s Modified Eagle Medium (DMEM) supplemented with 10% fetal bovine serum (FBS) and with 1% nonessential amino acids (Caco-2 and Huh-7.5 cells) or with 10% fetal bovine serum, 90 µg/ml streptomycine, 40 µg/ml penicillin, 44 mM sodium bicarbonate and 1 mM sodium pyruvate (Calu-3) at 37 °C and 5% CO_2_. Primary human vaginal epithelial cells (ATCC PCS-480-010) were cultured in epithelial mammary cell basal media supplemented with 5 μg/ml insulin, 0.004 ml/ml bovine pituitary extract, 10 ng/ml epidermal growth factor, and 0.5 μg/ml hydrocortisone (PromoCell. GmbH, Germany).

The HSV strains included HSV-1 (KOS), HSV-2(G), and HSV-1 K26GFP, which expresses green fluorescent protein fused to the viral capsid protein VP26^13^. The VSV viruses included native VSV (Indiana) and VSV viruses engineered to express enhanced GFP and either VSV glycoprotein G (VSV-G), Ebola virus glycoprotein (VSV-EBOV-GP) or SARS-CoV-2 spike (VSV-S) and were provided by K. Chandran, Albert Einstein College of Medicine) and propagated and titered on Vero cells^16,17^. SARS-CoV-2, strain USA-WA1/2020, was obtained from BEI Resources and amplified in Caco-2 cells. Caco-2 cells were infected at a MOI = 0.05 PFU/cell and incubated for 6 days at 37 °C. The virus-containing supernatant was subsequently harvested, clarified by centrifugation (3000×*g* × 10 min) and stored at −80 °C. Viral titers were measured on Huh-7.5 cells by standard plaque assay. Briefly, 500 µL of serial 10-fold virus dilutions in Opti-MEM were used to infect 4 × 10^5^ cells seeded the day prior into wells of a 6-well plate. After 90 min adsorption, the virus inoculum was removed, and cells were overlayed with DMEM containing 10% FBS with 1.2% microcrystalline cellulose (Avicel). Cells were incubated for 4 days at 33 °C, followed by fixation with 7% formaldehyde and crystal violet staining for plaque enumeration.

### Cytotoxicity and apoptosis assays

To assess effects of CIMSS on cell growth and viability, cells were plated in 96-well plates and allowed to adhere overnight to ~50% confluence (growth) or to 90–100% confluence (cytotoxicity) and then exposed to culture media alone, media containing increasing concentrations CIMSS or staurosporine or an equivalent concentration of DMSO (0.1% DMSO for 10 μM CIMSS or staurosporine, 0.5% for 50 μM, and 1% DMSO for 100 μM). Cell proliferation and viability were determined using the Cell Titer 96 Aqueous One Solution and optical density was determined using a SpectraMax M5e Molecular Devices multidetection microplate reader. For apoptosis assays, cells were grown on glass coverslips in 24-well plates and then exposed to 0.1% DMSO, CIMSS (10 µM) or staurosporine (10 µM) for 6 and 24 h. The cells were then fixed and stained for activated caspases (red) and SYTOX Green (for integrity of plasma membrane) by Image-iT^TM^ LIVE red Poly Caspase Detection kit. Nuclei were stained with Hoechst. Images were acquired by laser confocal microscope ZeissLive/DuoScan equipped with oil immersion objectives 63 × 1.1 and captured in an optical slice of 0.37 µm with appropriate filters. Alternatively, immunoblots were prepared and probed with antibodies to cleaved PARP-1 or cleaved caspase 8.

### HSV plaque, binding and entry assays

For plaque assays, HaCat cells were exposed to the indicated multiplicity of infection (MOI) for 1 h at 37 °C in the presence of DMSO, CIMSS or staurosporine, washed once with low pH buffer to inactivate extracellular virus and then three times with phosphate buffered saline (PBS, pH 7.4), before culturing in serum free media. After 48 h incubation, plaques were counted by immunoassay using an anti–human IgG antibody peroxidase conjugate (PA1-86064, Thermo Fisher)^33^. To evaluate the effects of drugs on viral binding, HaCat cells were exposed to escalating MOI of dextran-purified virus in the absence or presence of CIMSS for 4 h at 4 °C. Western blots were prepared and probed for gD as a marker of bound virus and β-actin as a control^34^.

To quantify viral entry, cells were synchronously infected by allowing virus to bind to cells at 4°C for 4 h, washed, and then transferred to 37 °C. CIMSS or control DMSO was added at the time of temperature shift. At indicated times post-temperature shift, the cells were treated with a low pH buffer to inactivate virus that had not penetrated, washed, overlaid with medium containing methyl cellulose and viral plaques were counted at 48 h. Entry was also assessed by immunoblotting nuclear extracts for VP16. HaCat or primary vaginal epithelial cells were mock-infected or infected with HSV-2(G) (MOI 10 pfu/cell) at 4 °C for 60 min, washed three times with cold PBS and overlaid with medium supplemented with DMSO (0.1%), CIMSS (10 µM), rabbit-anti-human Akt IgG (2 µg/ml), or control IgG (2 µg/ml). Nuclear extracts were prepared after 1 h and analyzed by western blot with antibodies targeting VP16, histone H1 and golgin-97^5^. The non-nuclear fraction was also probed for golgin-97 as a positive control. Additionally, HaCat cells were mock-infected or synchronously infected with HSV-1 K26GFP and, at time of temperature shift, treated with 10 μM CIMSS, 2 μg/ml polyclonal anti-Akt antibody, 10 μg/ml cycloheximide (company), or 0.1% DMSO. After 1 or 4 h of incubation, the cells were fixed. Plasma membranes were stained using Image -IT^TM^ LIVE Plasma Membrane kit and nuclei were stained with DAPI (Thermo Fisher Scientific, Cat 134406). The percentage of GFP + nuclei was determined by counting ~200 cells over 3–4 fields. Images were obtained with Leica SP8 microscope (objective 63 × 1.4); xz images were captured with optical slice 0.6 µm, 25–30 slices per image.

### VSV pseudotyped virus infection assays

Infection of Vero or Huh7 cells by VSV-G, VSV-EBOV-GP, and VSV-S was monitored by plaque assay after 48 h culture and staining with crystal violet. Calu-3 cells were infected with virus and after 96 h culture, supernatants were harvested and viral yields quantified by titering on Vero cells.

### SARS-CoV-2 assays

The day prior to infection Huh-7.5 cells were seeded into 96-well plates at two densities: 1.25 × 10^4^ cells/well and at 5 × 10^3^ cells/well for fixation at 24 hpi and at 72 hpi, respectively. The next day, serially diluted CIMSS (or DMSO control) was added to the wells, followed by infections with SARS-CoV-2 at MOIs of 0.25 PFU/cell (24 h timepoint) and 0.01 PFU/cell (72 h timepoint). Cells were then incubated at 37 °C for 24 h and at 33 °C for 72 h. At the respective timepoints, cells were fixed by adding an equal volume of 7% formaldehyde to the wells and subsequently permeabilized with 0.1% Triton X-100 for 10 min. After extensive washing, SARS-CoV-2 infected cells were incubated for 1 h at room temperature with blocking solution of 5% goat serum in PBS (catalog no. 005–000-121; Jackson ImmunoResearch). A rabbit polyclonal anti-SARS-CoV-2 nucleocapsid antibody (catalog no. GTX135357; GeneTex) was added to the cells at 1:1000 dilution in blocking solution and incubated at 4 °C overnight. Goat anti-rabbit AlexaFluor 594 (catalog no. A-11012; Life Technologies) was used as a secondary antibody at a 1:2000 dilution. Nuclei were stained with Hoechst 33342 (catalog no. 62249; Thermo Scientific) at a 1 µg/mL dilution. Images were acquired with a fluorescence microscope and analyzed using ImageXpress Micro XLS (Molecular Devices, Sunnyvale, CA). All SARS-CoV-2 experiments were performed in a biosafety level 3 laboratory.

### Antibodies and chemical reagents

Primary antibodies and dilutions were as follows: mouse anti-PtdS mAb, 1:200 (05-719, Millipore, Upstate Biotechnology, Lake Placid, NY); mouse anti-PLSCR1 mAb, 1:200 (ab24923, Abcam Cambridge, MA), rabbit anti-PLSCR1, 1:500 (NBP1-322588, NOVUS Biologicals, Littleton, CO); mouse anti-PLSCR1 (sc-27779, Santa Cruz Biotechnology); mouse anti-phosphotyrosine mAb (4G10; 05-1050X, Millipore,); rabbit anti-FIC1, 1:500 (sc-134967, Santa Cruz Biotechnology), mouse anti-β-actin mAb, 1:5000 (A-5441, Sigma-Aldrich); rabbit anti-phospho-Akt (Ser-473) mAb, 1:500 (4060 T, Cell Signaling Technology, Danvers, MA); rabbit anti-phospho-Akt (Thr-308) mAb, 1:500 (9275, Cell Signaling Technology); rabbit anti-Akt123, 1:1000 (sc-8312, Santa Cruz Biotechnology); rabbit anti-Akt,1:200 (9272S, Cell Signaling); rabbit anti-Akt1, 1:200 (SAB450007, Sigma Aldrich); rabbit anti-pan-Akt (phosphoT308),1:250 (ab38449, Abcam); rabbit anti-PDPK1,1:200 (3062S, Cell Signaling); rabbit anti-pPDPK1(S241), 1:300 (3438S, Cell Signaling); rabbit anti-pPLCγ1,1:300 (07-506, Upstate); mouse anti-PLCγ1, 1:200 (sc-374467, Santa Cruz Biotechnology); rabbit anti-cleaved caspase 8 (Asp374) (18C8), 1:1000 (9496, Cell Signaling Technology); rabbit anti-cleaved PARP-1 (Asp 214) (D64E10) XP, 1:1000 (5625, Cell Signaling Technology); mouse anti-ACE2, 1:100 (sc-390851, Santa Cruz Biotechnology), mouse anti-TPMRSS2, 1:100 (sc-51572, Santa Cruz Biotechnology), human anti-SARS-CoV2 Spike protein, 1:100 (703973, Invitrogen); mouse anti-HSV gD, 1:100 (HA025, Virusys Corporation, Taneytown, MD); goat anti-HSV VP16, 2 μg/ml (sc-17547, Santa Cruz Biotechnology); mouse anti-histone H1, 2 μg/ml (sc-8030, Santa Cruz Biotechnology); mouse anti-golgin97 (sc-59820, Santa Cruz Biotechnology); anti-goat IgG, 1:500 (STAR AR122, Bio-Rad), control mouse IgG, 1:100 (sc-2025, Santa Cruz Biotechnology, goat anti-β-actin (Thermo Fisher Scientific), and rabbit anti-GFP, 1:250 (ab32146, Abcam). Annexin V Alexa Fluor 555 was purchased from Thermo Fisher Scientific (A 35108) The secondary antibodies for Western blots were horseradish peroxidase-conjugated goat anti-mouse (170-5047, Bio-Rad, Hercules, CA), goat anti-rabbit (170-5046, Bio-Rad), and donkey anti-goat 1:1000 (sc-2020, Santa Cruz). The secondary antibodies for confocal microscopy were goat anti-mouse Alexa Fluor 350 (A-11045, Invitrogen Molecular Probes), goat anti-mouse Alexa Fluor 555 (A-21147, Thermo Fisher), goat anti mouse Alexa Fluor 488 (A11001, Thermo Fisher) and goat anti-rabbit Alexa Fluor 488 (A-11078, Thermo Fisher) or Alexa Fluor 555 (A-21428, Thermo Fisher). All secondary antibodies were diluted 1:1000. Staurosporine (PHZ1271) was purchased from Invitrogen Molecular Probes and apyrase from New England BioLabs (MO398S). Cell Titer 96 Aqueous One solution Cell proliferation Assay” was purchased from Promega (G3580 Promega, San Luis Obispo, CA, USA), Image-iT^TM^ LIVE red Poly Caspases Detection kit was purchased from Invitrogen (I35101, Thermo Fisher Scientific). Human recombinant insulin was purchased from MP Biomedicals.

### Confocal microscopy

Cells were grown on glass coverslips in 12- or 24-well plates and treated with 10 μM insulin in the absence or presence of CIMSS (10 or 100 µM) or staurosporine (0.1, 1, or 10 µM), fixed with 4% paraformaldehyde solution (Electron Microscopy, Hatfield, PA, USA) with or without permeabilization with 0.1% Triton X-100 and stained with conjugated antibodies to detect total or phosphorylated Akt (pAkt^t308^) and with DAPI (D1306, Invitrogen) to detect nuclei. In other experiments, cells were exposed to indicated viruses in the absence or presence of drug (CIMSS, staurosporine or DMSO control) and at different times post-viral exposure fixed with or without permeabilization. To label plasma membranes cells were incubated with blue-fluorescent Alexa Fluor 350 wheat germ agglutinin (I34406, W11261, Invitrogen Molecular Probes, Carlsbad, CA, USA) before infection. Conjugated antibodies to detect other cellular proteins are noted above (see Antibodies and chemical reagents). Images were acquired by laser confocal microscope ZeissLive/DuoScan equipped with oil immersion objectives 63 × 1.4 and 100 × 1.4 or Leica SP8 equipped with oil immersion objectives 63 × 1.4, captured in an optical slice of ~0.37 µm with appropriate filters. Alexa Fluor 488 and GFP were excited using the 488-nm line of a krypton/argon laser and viewed with a 505- to 530-nm band pass µm; AlexaFluor 350 was excited with 405-nm diode laser and collected with 420 to 475 nm filter; AlexaFluor 555 was excited using 561-nm helium/neon laser and collected with a 575 to 655 filter. All images were captured using the multitrack mode of the microscope to decrease cross talk of fluorescent signals (Zeiss LSM); 3D and extended focus images were generated using Volocity 5.3 software (Improvision, Lexington, MA). Images on Leica Sp8 were captured using excitation lines 405 nm, Argon (458 nm, 476 nm, 488 nm, 496 nm, 524 nm), collected by adjustable emission windows. All images were captured using one HyD, two PMTs (photomultipliers) and processed by LAS X. The number of GFP, PLSCR1, Akt, PtdS, PDPK1, and PLC γ1 positive cells was quantified using Cell Counter ImageJ software (NIH).

### Western blots

Cells were serum-starved for 24 h and then exposed to HSV-2(G) (MOI = 10 PFU/cell) in the presence of control buffer (0.1% DMSO) or CIMSS (10 µM). At different times post-viral exposure, the cells were harvested and lysed in buffer containing 20 mM Tris pH 7.5, 50 mM NaCl, 1% NP-40, 0.05% DOC, supplemented with fresh protease and phosphatase inhibitors (118735, Roche Diagnostics, and P0044, P5726, Sigma Aldrich, respectively). Proteins were separated by SDS-PAGE and transferred to membranes for immunoblotting with the indicated antibodies. Blots were visualized, scanned and the band intensities were analyzed using ChemiDoc imaging system equipped with GelDoc2000 software (RRID:SCR_014210, Bio Rad). Western blots were quantified using ImageJ software (NIH).

### Biotinylation of cell surface proteins

Cells were exposed to HSV-2 (MOI = 10 PFU/cell) or mock infected in the presence of control buffer (0.1% DMSO) or CIMSS (10 µM) for 15 and 30 min, washed four times with ice-cold PBS and biotinylated with sulfo-NHS-SS-Biotin (F20650; Invitrogen Molecular Probes) for 1 h at 4 °C. After three washes with PBS supplemented with 1% BSA, cells were harvested, solubilized in PBS containing a proteinase inhibitor cocktail, precipitated with streptavidin magnetic beads (Dynabeads M-280 Streptavidin; Life Technologies, Gaithersburg, MD, USA) and analyzed by immunoblotting.

### Calcium kinetic measurements

HaCat cells (5 × 10^4^) were seeded in 96-well black plates with clear bottoms (3340, CellBIND surface, Corning Inc., NY) and incubated with 25 μM Fura-2 AM diluted in PBS (F1221, Invitrogen Molecular Probes) for 60 min at 37 °C, rinsed with PBS thrice, placed on ice and then exposed to cold dextran gradient purified HSV-2 (5 pfu/cell) or control buffer (PBS)^5^. The cells were then transferred to SpectraMaxMF^e^ temperature-regulated chamber at 37^o^C (Molecular Devices Ca) without washing; photometric data for intracellular Ca^2+^ concentration [Ca^2+^] were generated by exciting cells at 340 and 380 nm and measuring emission at 510 nm every minute for one hour using SoftMaxPro. 5.4 software (Molecular Devices). An intracellular calibration was performed with each experiment by determining the fluorescence ratio (340:380) in the presence of Ca-free 10 mM K_2_EGTA buffer (*R*_min_) and 10 mM CaEGTA buffer containing 10 µM ionomycin (*R*_max_) (C-3008, Calcium Calibration Buffer Kit #1, Invitrogen Molecular Probes). The mean [Ca^2+^] was determined from four wells according to the manufacturer’s recommendations using the following equation: [Ca^2+^]= *K*_d_ Q (*R*−*R*_min_)/(*R*_max_−*R*), where *R* represents the fluorescence intensity ratio *F*_λ1_/*F*_λ2_; *λ*1(340 nm) and *λ*2 (380 nm) are the fluorescence detection wavelengths for ion-bound and ion-free indicators; *K*_d_ is the Ca^2+^ dissociation constant and equals 0.14 µM (Fura and Indo Ratiometric Calcium Indicators, Invitrogen Molecular Probes); and *Q* is the ratio of *F*_min_ to *F*_max_ at *λ*2 (380 nm).

### Immunoprecipitation assays

Cells were serum-starved for 24 h prior to being exposed to HSV-2(G) (MOI = 10 PFU/cell) in the presence of control buffer (0.1% DMSO) or CIMSS (10 µM for 30 min, washed theee times with PBS, and then immediately placed on ice. The cells were then lysed by sonication in RIPA buffer (Thermo Scientific) supplemented with complete protease inhibitors (Roche Diagnostics). The lysates were incubated overnight at 4 °C with rabbit anti-PLSR1 and then immune complexes were isolated following a 4 h incubation with protein G Plus agarose beads (sc-500778, Santa Cruz Biotechnology). The precipitated complexes (pellet), supernatants or an aliquot of the cell lysate were analyzed by Western blot with mouse anti-scramblase mAbs or with mouse anti-phosphotyrosine mAb. (mPY).

### Small interfering RNA (siRNA) transfections

Cells were transfected with 10 nM of the indicated siRNA sequences in 12-well plates using the HiPerFect Transfection Reagent (1029975, Qiagen). Akt1 siRNA (sc-29195) was purchased from Santa Cruz Biotechnology (Santa Cruz, CA, USA) and a control siRNA (Cat# AM4636) was purchased from Applied Biosystems (Applied Biosystems, Foster City, CA). Cells were analyzed for protein expression by preparing Western blots of cell lysates 72 h post-transfection.

### Statistics and reproducibility

Analyses were performed using GraphPad Prism version 9.0 software (GraphPad Software Inc. San Diego, CA). A *p* value of 0.05 was considered statistically significant. Results were compared using unpaired Student’s *t* tests or one-way ANOVA with correction for multiple comparisons as indicated. The number of biological and technical replicates is indicated for each figure and presented as dot blots to show data distribution.

### Reporting summary

Further information on research design is available in the Nature Research Reporting Summary linked to this article.

## Supplementary information

Supplementary Information

Description of Additional Supplementary Files

Supplementary Data 1

Supplementary Data 2

Reporting Summary

## Data Availability

Data supporting the findings of the study are available within the article and in Supplementary Information and Supplementary Data 1 and Data 2 files. All other data are available from the corresponding authors upon request.
