# Author Correction: Cell-impermeable staurosporine analog targets extracellular kinases to inhibit HSV and SARS-CoV-2

**DOI:** 10.1038/s42003-023-05207-0

**Published:** 2023-08-10

**Authors:** Natalia Cheshenko, Jeffrey B. Bonanno, Hans-Heinrich Hoffmann, Rohit K. Jangra, Kartik Chandran, Charles M. Rice, Steven C. Almo, Betsy C. Herold

**Affiliations:** 1 Department of Pediatrics, Albert Einstein College of Medicine, Bronx, NY USA; 2 Department of Biochemistry, Albert Einstein College of Medicine, Bronx, NY USA; 3 Laboratory of Virology and Infectious Disease, The Rockefeller University, New York, NY USA; 4 Department of Microbiology and Immunology, Albert Einstein College of Medicine, Bronx, NY USA; 5 Present Address: Department of Microbiology and Immunology, Louisiana State University Health Science Center-Shreveport, Shreveport, LA USA

**Keywords:** Herpes virus, Kinases

Correction to: *Communications Biology* 10.1038/s42003-022-04067-4, published online 16 October 2022.

The original version of the Article contained an error in Figure 1a showing the structure of the compound CIMSS.

The original article has been corrected.

