# Supplementary material for: Cell-impermeable staurosporine analog targets extracellular kinases to inhibit HSV and SARS-CoV-2
Source: Commun Biol. 2022 Oct 16;5:1096. doi: 10.1038/s42003-022-04067-4 (PMC9569420; doi:10.1038/s42003-022-04067-4)

Supplementary Information

Cell-Impermeable Staurosporine Analog Targets Extracellular Kinases to Inhibit HSV and SARS-CoV-2

Natalia Cheshenko[1], Jeffrey B. Bonanno[2], Hans-Heinrich Hoffmann[3], Rohit K. Jangra[4†], Kartik Chandran[4], Charles M. Rice[3], Steven C. Almo[2#] and Betsy C. Herold[1,4#]

Department of Pediatrics, Albert Einstein College of Medicine, Bronx NY USA[1], Department of Biochemistry, Albert Einstein College of Medicine, Bronx, NY, USA[2], Laboratory of Virology and Infectious Disease, The Rockefeller University, New York, NY, USA[3], Department of Microbiology and Immunology, Albert Einstein College of Medicine, Bronx, NY, USA[4]

Corresponding author: Betsy C. Herold, MD, Albert Einstein College of Medicine, 1225 Morris Park Avenue, VE6A03, Bronx, NY 10461 betsy.herold@einsteinmed.edu 718-839-7460

[#]These authors jointly supervised the project (steve.almo@einsteinmed.edu)

[†]Current address: Department of Microbiology and Immunology, Louisiana State University Health Science Center-Shreveport, Shreveport, LA, USA.

Supplementary Table 1

In vitro kinase 50% Inhibitory Concentration (IC_{50}) for CIMSS and Staurosporine

	IC50 (Molar concentration)	
**Kinase**	**CIMSS**	**Staurosporine**
ABL1	1.43E-07	2.22E-08
AKT1	4.61E-07	2.00E-09
AKT2	1.00E-06	1.42E-08
AKT3	1.57E-07	1.54E-09
c-Src	1.19E-08	1.61E-09
EGFR	1.61E-06	9.41E-08
Insulin Receptor	8.03E-07	1.86E-08
PDPK1	2.78E-10	5.10E-10
PKA	4.23E-08	9.12E-10
PKCd	3.64E-09	1.27E-10

Supplementary Table 2.

Permeability studies for CIMSS relative to control compounds

**MDCK Transport***			
**Compound**	**Conc. [µM]**	**Papp**	**Recover %**
Digoxin	5	3.1	96
Propranolol	5	67.4	93
CIMSS	5	0.1	96
**Parallel artificial membrane permeability assay (PAMPA)****			
Propranolol	100	8.991	85
Atenolol	100	0.001	96
CIMSS	100	0.005	83

*The apparent permeability coefficient (Papp) was calculated as the average of two measurements $x10^{-6}$ cm/s
**PAMPA was calculated as the average of four measurements $x10^{-6}$ cm/s

Supplementary Figure 1: Western blots for Figure 2c. HaCat cells were exposed to 0.1% DMSO, 0.1, 1 or 10 μM CIMSS or 0.01, 0.1, 1 or 10 μM staurosporine and after 8 hours of incubation, lysates were prepared and analyzed by Western blotting for cleaved PARP-1(a) or cleaved caspase 8 (b). Blots were also probed for β-actin (c and d). Blue boxes indicate cropped sections shown in main text for all blots.

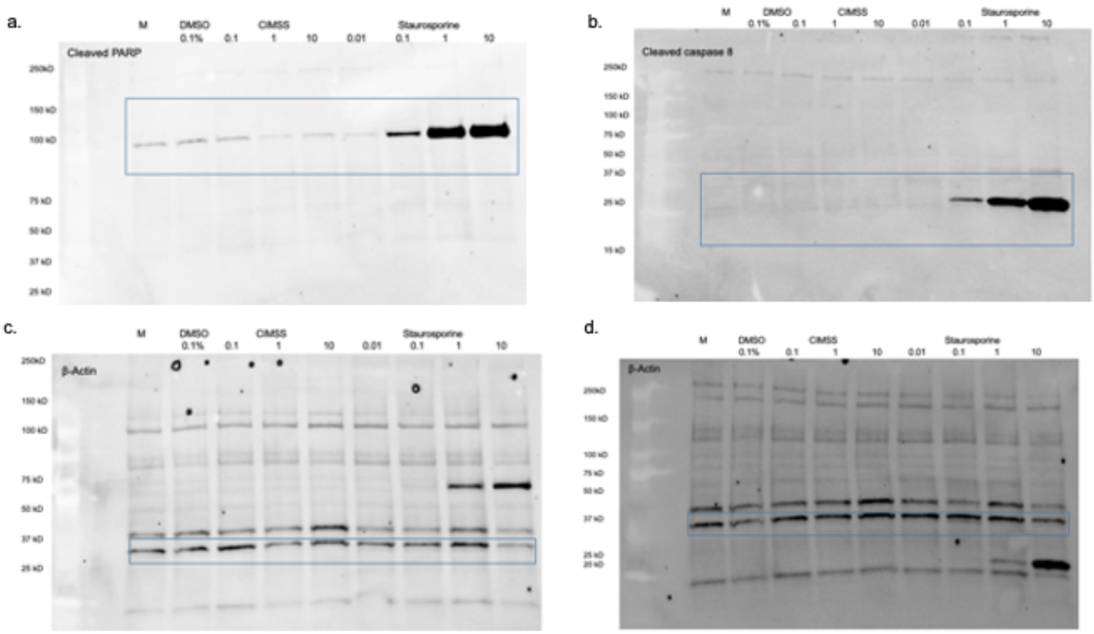

Supplementary Figure 2: Western blots for Figure 3c. HaCat or primary vaginal epithelial cells were mock or synchronously infected with HSV-2(G) (MOI=10 pfu/cell) and 0.1% DMSO, 10 μM CIMSS, 2 μg/ml rabbit anti-Akt or a control IgG were added at the time of temperature shift. Nuclear extracts were prepared after 1 h incubation at 37°C and probed with antibodies for VP16, histone-1 (nuclear protein) or Golgin-1 (cytoplasmic protein).

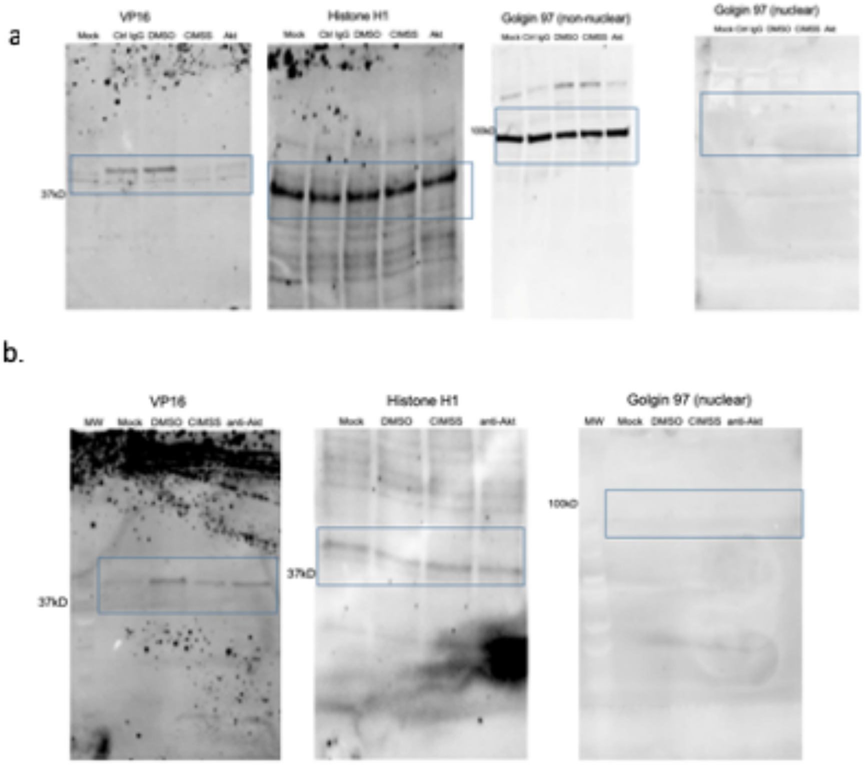

Supplementary Figure 3. CIMSS and anti-Akt antibodies inhibit HSV capsid entry. HaCat cells were synchronously infected with HSV-1K26GFP, which expresses a green fluorescent protein fused to the capsid protein VP26 (MOI 10 PFU/cell), with the addition of 0.1% DMSO, 10 μM CIMSS, 2 μg/ml polyclonal anti-Akt antibody or 10 μg/ml of cycloheximide at the time of temperature shift. After 1 or 4 hours of incubation, the cells were fixed. Plasma membranes were stained using Image -IT™ LIVE Plasma Membrane kit and nuclei were stained with DAPI. The percentage of GFP+ cells was determined by counting ~200 cells over 3-4 fields. Images were obtained with Leica SP8 microscope, objective 63x1, bar=18μm; xz images were captured with optical slice of 0.6μm, 25-30 slices per image (***$p < 0.001$ and ****$p < 0.0001$ compared to DMSO, ANOVA).

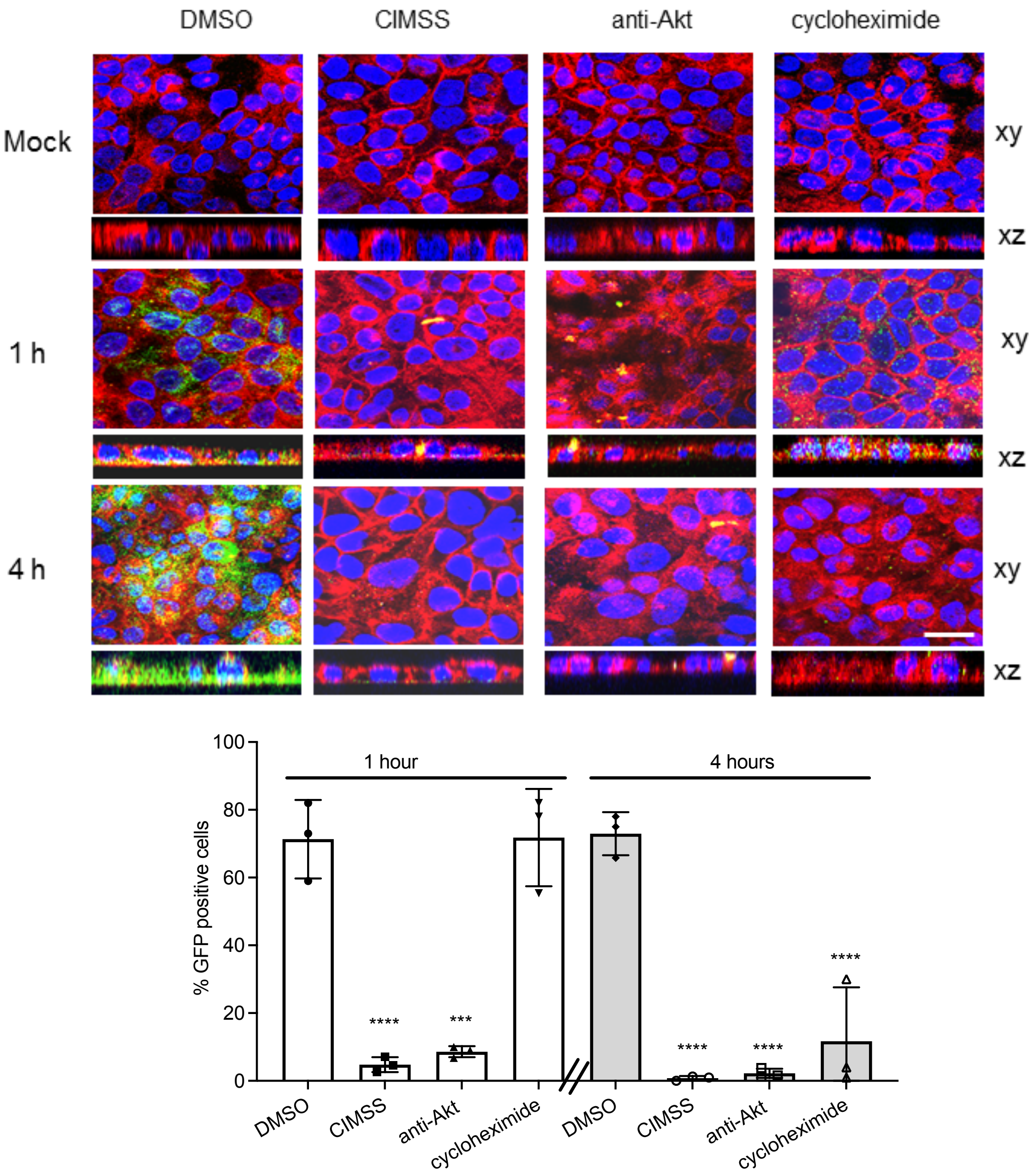

Supplementary Figure 4: Western blots for Figures 4a and 4c.

(4a) HaCat cells were exposed to HSV-2(G) at the indicated MOIs in the absence of presence of 10 μM CIMSS for 4 hours at 4°C. The cells were then washed, lysed and Western blots of cell lysates prepared and probed with a mAb to gD as a marker of cell-bound virus and anti-β-actin as a loading control.

(4c) HaCaT cells were mock-infected or infected with HSV-2(G) in the presence of 0.1% DMSO, 10 μM CIMSS or 10 μM staurosporine for 30 minutes and then the cells were lysed and incubated with rabbit anti-PLSCR1 antibody and immune complexes precipitated with protein A-agarose and analyzed by Western blotting with a mouse anti-phosphotyrosine (PY20) or mouse anti-PLSCR mAb.

Figure 4a

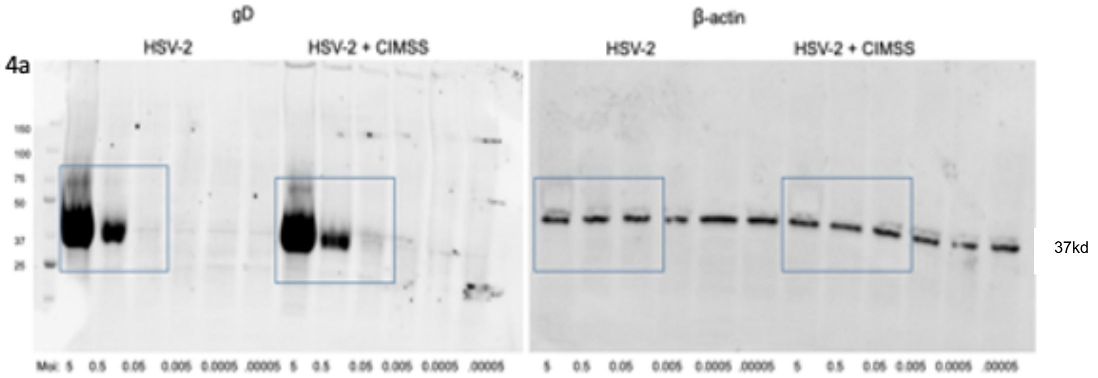

Figure 4c

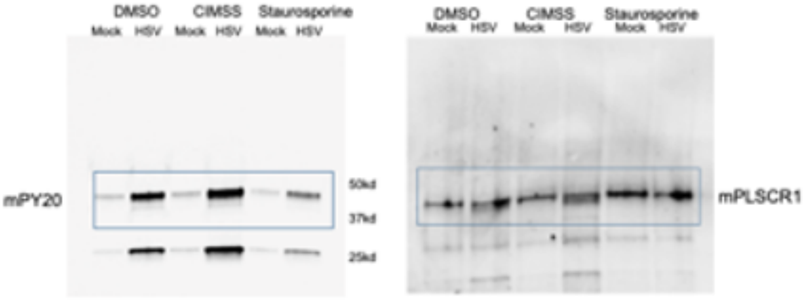

Supplementary Figure 5: Quantification of confocal images. Confocal images were scanned and the mean fluorescence intensity of phosphatidylserines and Akt from **Figure 4d** (a), phosphorylated Akt ($pAkt^{S473}$ and $pAkt^{T308}$) from non-permeabilized and permeabilized cells from **Figure 4d** (b) and phosphatidylserines from **Figure 8b** (c) were quantified after counting 200-300 cells over 4 independent fields.

a.

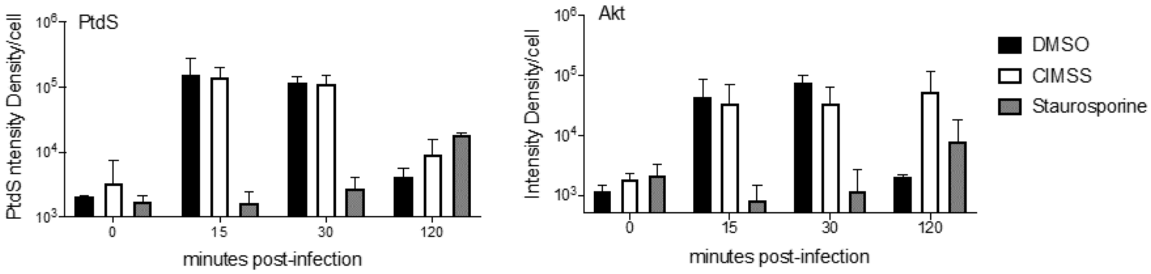

b.

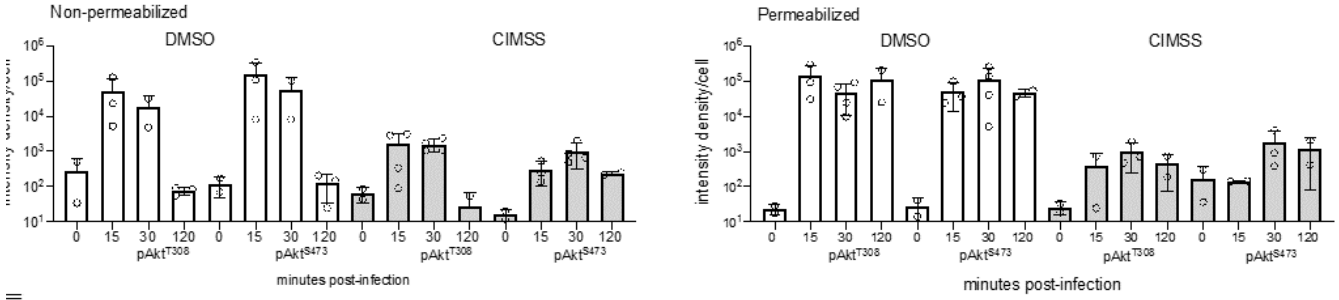

c.

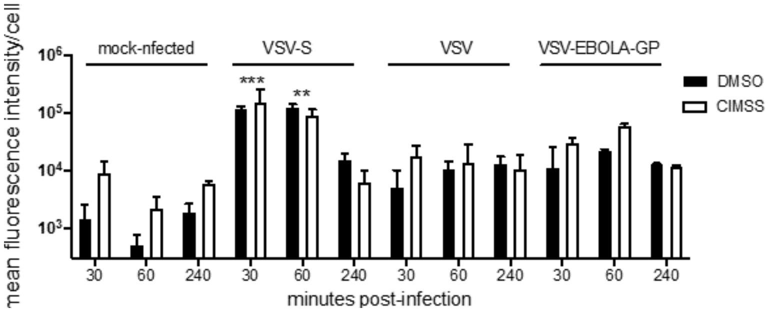

Supplementary Figure 6: Immunoblots corresponding to Figure 5a. HaCat cells were mock-infected or synchronously infected with HSV-2(G) in the absence or presence of 10 μM CIMSS. After 15 minutes incubation, cell surface proteins were biotinylated and precipitated with streptavidin magnetic beads and analyzed by immunoblotting with Abs to pPDPK1^{S241} and total PDPK1 (a), pPLCγ1^{Y783} and total PLCγ1 (b), pAkt^{T308} and total Akt (c), and FIC-1 (cytosolic protein) (d). Controls include whole cell lysates.

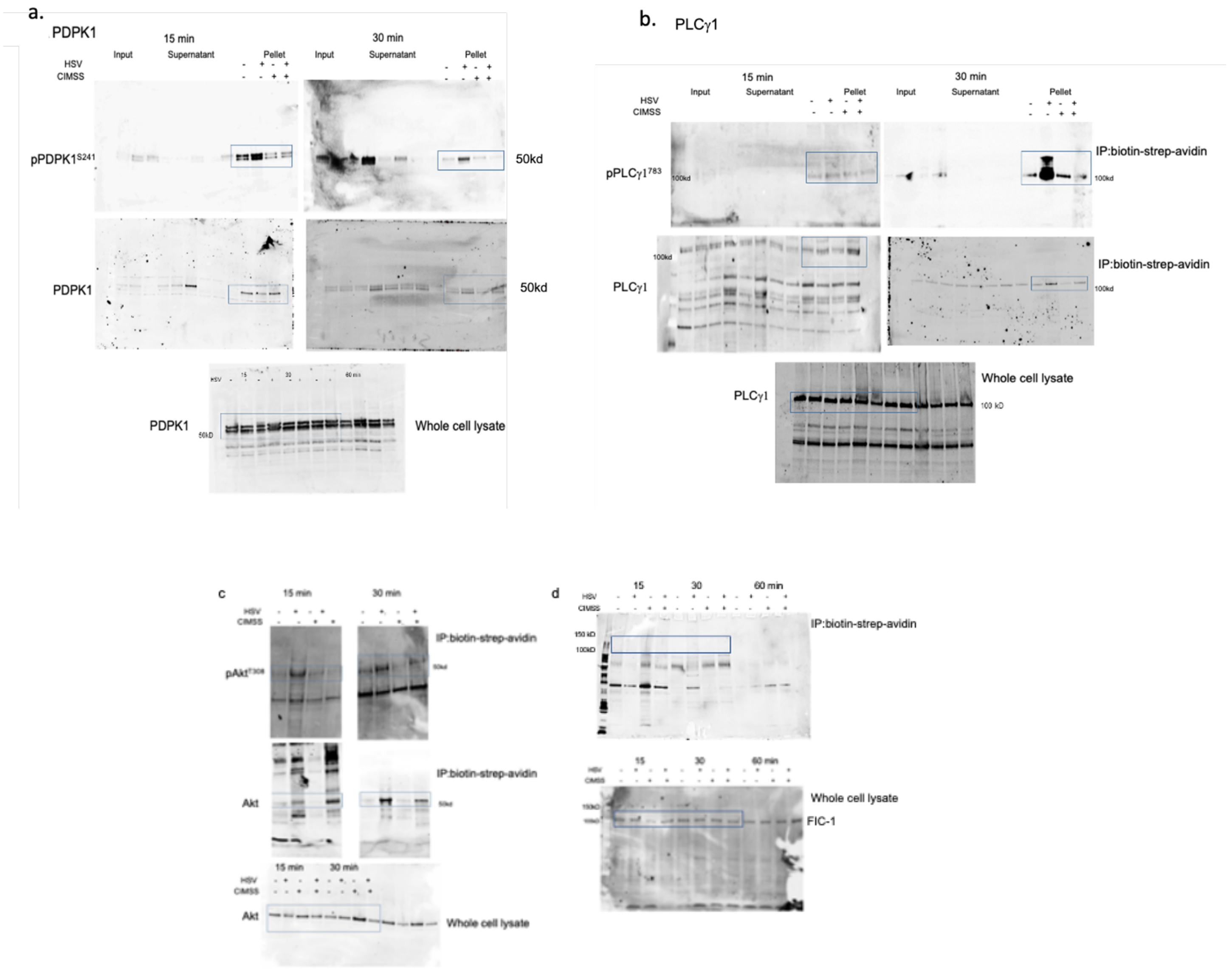

Supplementary Figure 7: Western blots corresponding to Figures 6a and Figure 7b.

a. HaCat cells were transfected with siControl (siCtrl), siAkt1, or siPDPK1 and silencing assessed by preparing Western blots after 72 h and probing for Akt, PDPK1 and β-actin as indicated.

b. Western blots of Vero or Huh7 cell lysates probed for TMPRSS2 and β-actin as loading control.

a.

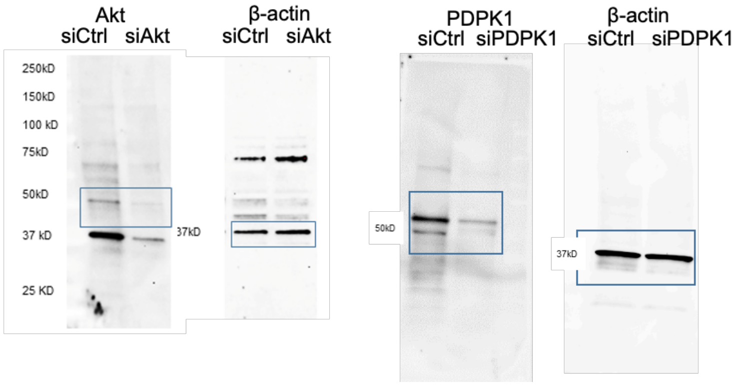

b.

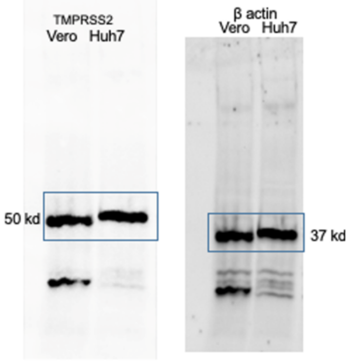

Supplementary Figure 8: Western blots corresponding to Figure 8a. Vero cells were mock-infected or infected (a, c) with VSV pseudotyped with Spike (a, c), VSV-G (b, d), or EBOV-GP (b,d) for 30 minutes in the absence or presence of CIMSS or staurosporine (10 μM each) or murine anti-ACE2 (anti-ACE), anti-Spike (anti-S) or an isotype control IgG (10 μg/ml of each immunoglobulin). The cells were lysed and incubated with rabbit anti-PLSCR1 antibody and immune complexes precipitated with protein A-agarose and analyzed by Western blotting with a mouse anti-phosphotyrosine (PY20) (a, b) or mouse anti-PLSCR mAb (c, d).

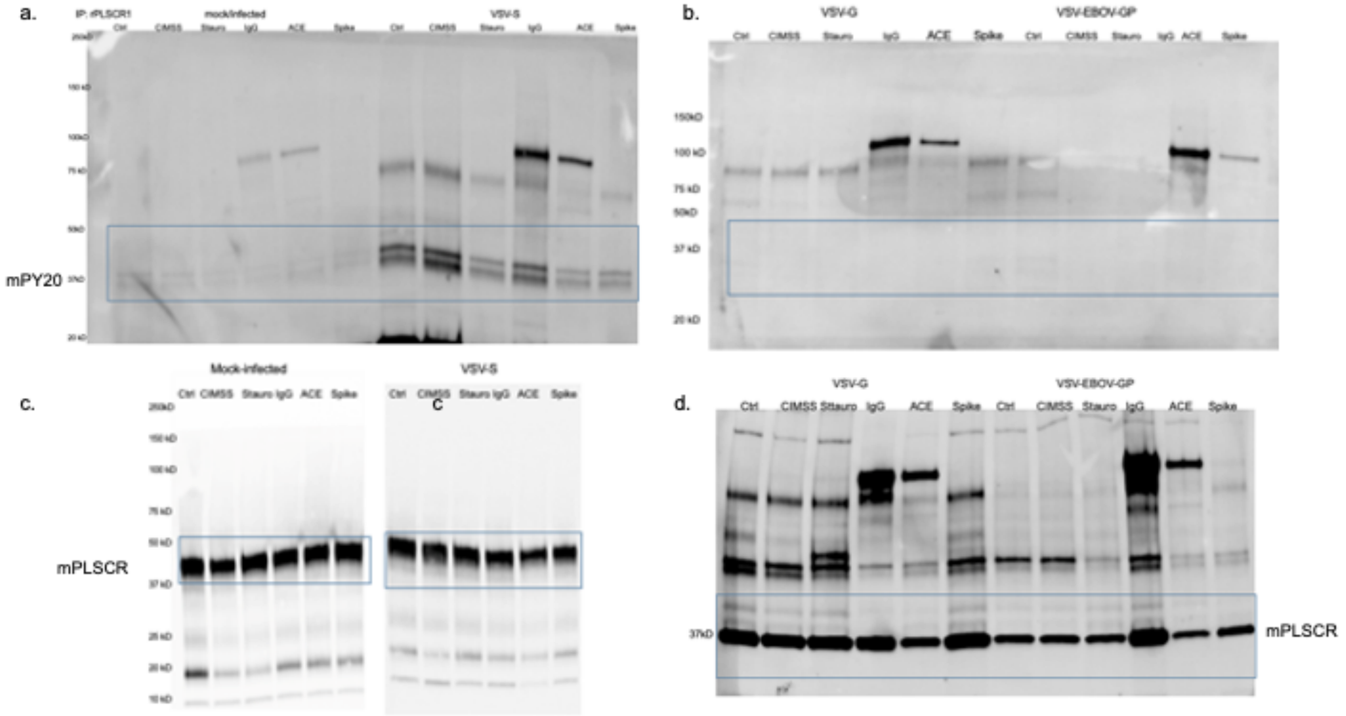

Supplementary Figure 9: Western blots corresponding to Figure 8c. Vero cells were mock-infected or infected with the indicated VSV pseudotyped viruses in the absence or presence of 10 µM CIMSS. After 15, 30 or 60 minutes, the cell surface proteins were biotinylated and precipitated with streptavidin magnetic beads and analyzed by immunoblotting with Abs to pAkt^{T308} (a and b) and total extracellular Akt (c and d).

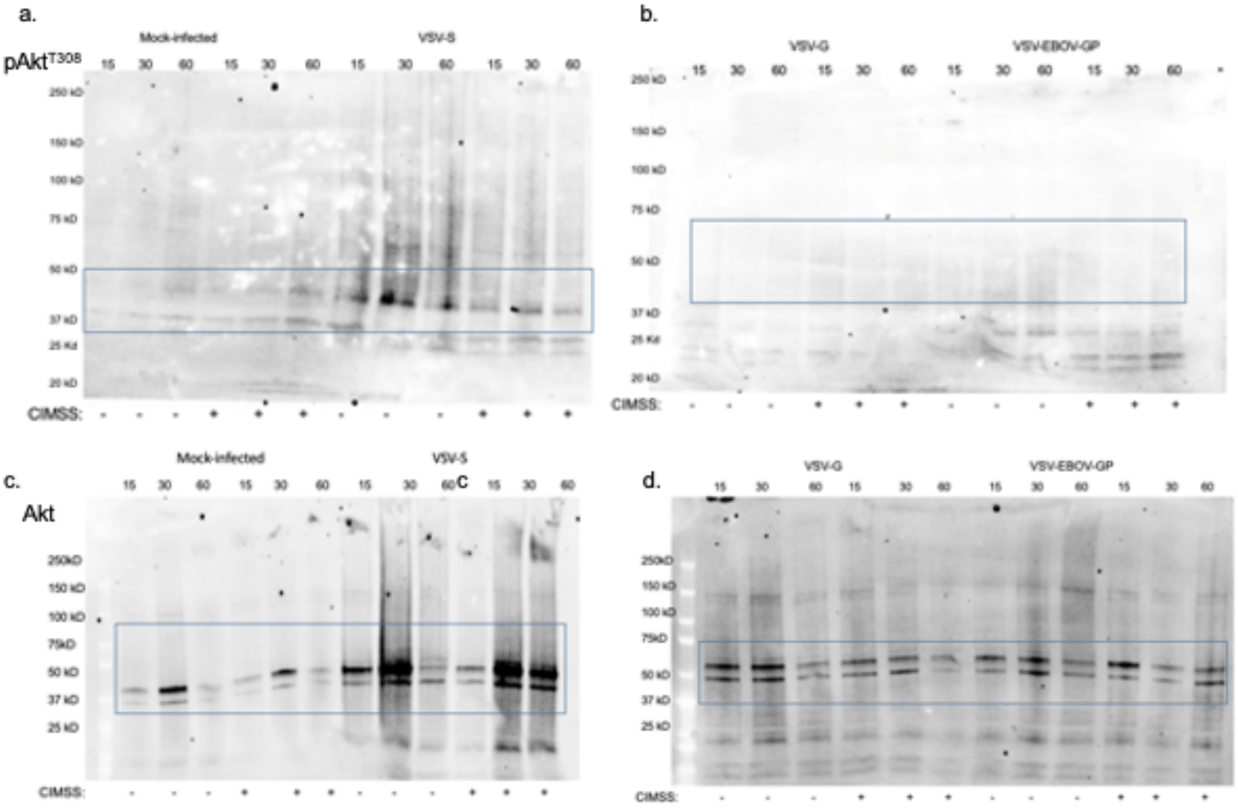

Supplementary Figure 10: VSV-Spike triggers translocation of Akt, PDP1 to the outer leaflet and the phosphorylation of these kinases is inhibited by CIMSS. Vero cells were infected with VSV-S or native VSV-G in the presence of 0.1% DMSO or 10 μM CIMSS and at baseline (t=0 minutes), 30 minutes and 60 minutes following infection, the cells were fixed with or without Triton X-100 permeabilization and stained with conjugated antibodies to phosphorylated Akt, phosphorylated PDPK1, total Akt or total PDPK1; nuclei were stained blue with DAPI. Results are representative of 2 independent experiments and were obtained with Leica SP8 microscope (objective 63x1.4).

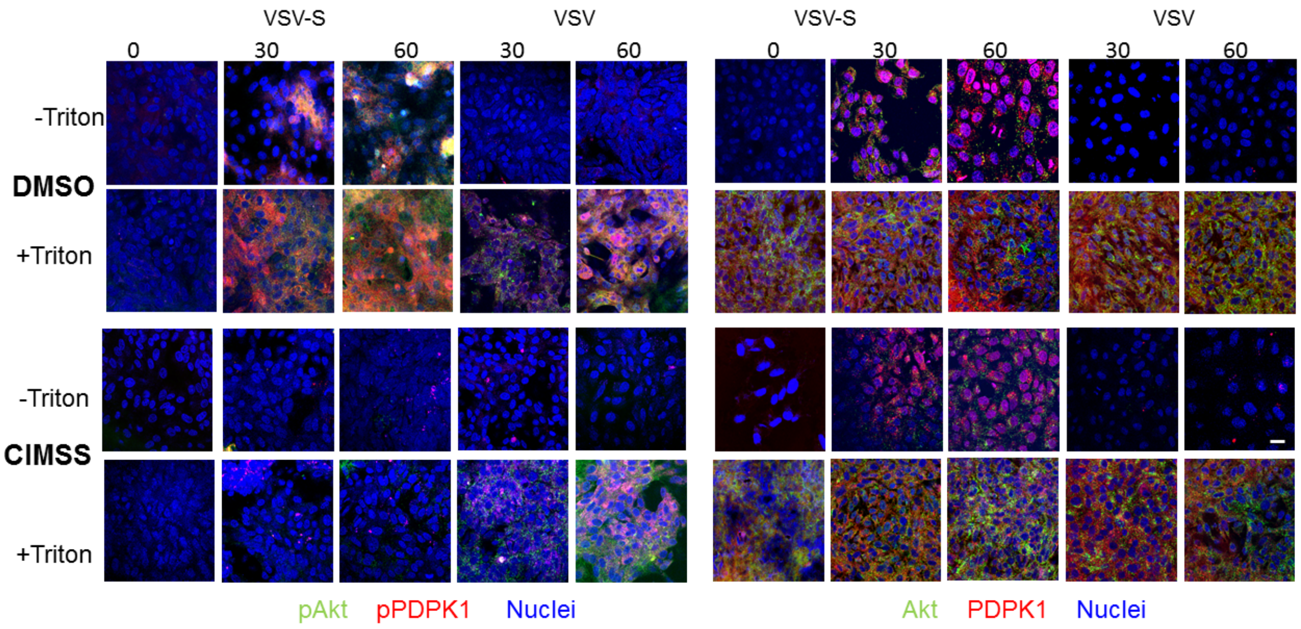

Supplementary Figure 11: Western blots corresponding to Figure 9a. Vero cells were transfected with control siRNA (siCtrl) or siRNA targeting Akt1, PDPK1, PLCγ or FIC-1 and after 72 h, cell lysates were assayed by preparing Western blots and probing for respective proteins. Cell lysates were probed for β-actin on separate blots.

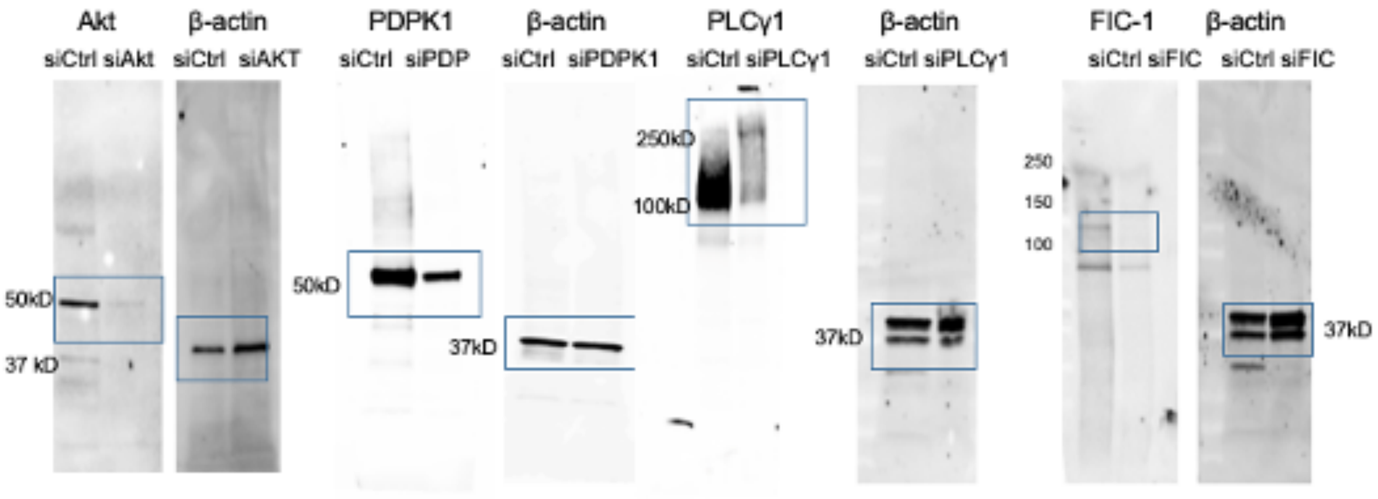

Supplement: Supplementary file 1 — Supplementary Information [file 42003_2022_4067_MOESM1_ESM.pdf]
