# Supplementary material for: Cell-impermeable staurosporine analog targets extracellular kinases to inhibit HSV and SARS-CoV-2
Source: Commun Biol. 2022 Oct 16;5:1096. doi: 10.1038/s42003-022-04067-4 (PMC9569420; doi:10.1038/s42003-022-04067-4)
Supplement: Supplementary file 2 — Description of Additional Supplementary Files [file 42003_2022_4067_MOESM2_ESM.pdf]

Description of Additional Supplementary Files

File name: Supplementary Data 1
Description: CIMSS (10 µM) activity against a panel of 393 kinases and compared to staurosporine or indicated controls.

File name: Supplementary Data 2
Description: Primary data for each manuscript figure.
