# Supplementary material for: Cell-impermeable staurosporine analog targets extracellular kinases to inhibit HSV and SARS-CoV-2
Source: Commun Biol. 2022 Oct 16;5:1096. doi: 10.1038/s42003-022-04067-4 (PMC9569420; doi:10.1038/s42003-022-04067-4)
Supplement: Supplementary file 3 — Supplementary Data 1 [file 42003_2022_4067_MOESM3_ESM.pdf]

Supplementary Data 1

Cell-Impermeable Staurosporine Analog Targets Extracellular Kinases to Inhibit HSV and SARS-CoV-2

Natalia Cheshenko[1], Jeffrey B. Bonanno[2], Hans-Heinrich Hoffmann[3], Rohit K. Jangra[4†], Kartik Chandran[4], Charles M. Rice[3], Steven C. Almo[2#] and Betsy C. Herold[1,4#]

Department of Pediatrics, Albert Einstein College of Medicine, Bronx NY USA[1], Department of Biochemistry, Albert Einstein College of Medicine, Bronx, NY, USA[2], Laboratory of Virology and Infectious Disease, The Rockefeller University, New York, NY, USA[3], Department of Microbiology and Immunology, Albert Einstein College of Medicine, Bronx, NY, USA[4]

Corresponding author: Betsy C. Herold, MD, Albert Einstein College of Medicine, 1225 Morris Park Avenue, VE6A03, Bronx, NY 10461 betsy.herold@einsteinmed.edu 718-839-7460

[#]These authors jointly supervised the project (steve.almo@einsteinmed.edu)

[†]Current address: Department of Microbiology and Immunology, Louisiana State University Health Science Center-Shreveport, Shreveport, LA, USA.

Supplementary Data 1

CIMSS (10 μM) activity against a panel of 393 kinases and compared to staurosporine or indicated controls

Kinase:	% Enzyme Activity (relative to DMSO controls) CIMSS (10μM)		IC_{50} (M) of Control Compound	Control compound
	Data 1	Data 2		
**ABL1**	5.18	4.92	**4.00E-08**	**Staurosporine**
**ABL2/ARG**	4.07	3.90	**1.19E-08**	**Staurosporine**
**ACK1**	7.33	5.60	**5.68E-08**	**Staurosporine**
**AKT1**	23.73	23.61	**7.80E-09**	**Staurosporine**
**AKT2**	29.76	19.09	**2.61E-08**	**Staurosporine**
**AKT3**	11.41	9.86	**1.77E-09**	**Staurosporine**
**ALK**	4.12	3.76	**3.17E-09**	**Staurosporine**
**ALK1/ACVRL1**	70.00	69.61	**1.31E-08**	**LDN193189**
**ALK2/ACVR1**	72.71	71.88	**1.78E-08**	**LDN193189**
**ALK3/BMPR1A**	79.51	79.43	**3.21E-08**	**LDN193189**
**ALK4/ACVR1B**	58.26	57.94	**1.02E-07**	**LDN193189**
**ALK5/TGFBR1**	86.58	85.77	**2.13E-07**	**LDN193189**
**ALK6/BMPR1B**	88.27	87.18	**1.22E-08**	**LDN193189**
**ARAF**	154.16	152.51	**1.39E-08**	**GW5074**
**ARK5/NUAK1**	1.83	1.45	**2.96E-09**	**Staurosporine**
**ASK1/MAP3K5**	6.25	6.05	**1.11E-08**	**Staurosporine**
**Aurora A**	1.92	1.08	**1.73E-09**	**Staurosporine**
**Aurora B**	2.85	2.83	**5.55E-09**	**Staurosporine**
**AURORA C**	2.59	2.18	**2.98E-09**	**Staurosporine**
**AXL**	3.61	3.52	**8.93E-09**	**Staurosporine**
**BLK**	0.34	0.29	**2.07E-09**	**Staurosporine**
**BMPR2**	50.39	49.89	**2.00E-07**	**Staurosporine**
**BMX/ETK**	2.19	1.32	**6.88E-09**	**Staurosporine**
**BRAF**	131.63	131.61	**7.62E-09**	**GW5074**
**BRK**	25.50	24.16	**1.74E-07**	**Staurosporine**
**BRSK1**	1.55	1.28	**4.15E-10**	**Staurosporine**
**BRSK2**	1.66	1.53	**2.53E-09**	**Staurosporine**
**BTK**	6.64	5.98	**3.00E-08**	**Staurosporine**
**c-Kit**	0.28	-0.14	**7.38E-10**	**Staurosporine**
**c-MER**	2.17	2.15	**1.68E-08**	**Staurosporine**
**c-MET**	12.40	9.84	**1.77E-07**	**Staurosporine**
**c-Src**	2.95	2.85	**3.33E-09**	**Staurosporine**
**CAMK1a**	28.74	28.61	**5.53E-09**	**Staurosporine**
**CAMK1b**	59.52	57.60	**4.15E-09**	**Staurosporine**
**CAMK1d**	20.31	19.69	**3.17E-10**	**Staurosporine**
**CAMK1g**	8.97	8.82	**3.21E-09**	**Staurosporine**
**CAMK2a**	1.36	1.34	**6.56E-11**	**Staurosporine**

**CAMK2b**	3.03	2.90	**5.53E-11**	**Staurosporine**
**CAMK2d**	1.33	0.88	**5.24E-12**	**Staurosporine**
**CAMK2g**	1.25	0.85	**3.45E-10**	**Staurosporine**
**CAMK4**	66.61	62.68	**1.32E-07**	**Staurosporine**
**CAMKK1**	1.00	1.00	**5.39E-08**	**Staurosporine**
**CAMKK2**	1.86	1.81	**7.53E-08**	**Staurosporine**
**CDC7/DBF4**	3.98	3.26	**1.47E-08**	**Staurosporine**
**CDK1/cyclin A**	21.33	18.54	**3.80E-09**	**Staurosporine**
**CDK1/cyclin B**	13.58	13.39	**2.32E-09**	**Staurosporine**
**CDK1/cyclin E**	17.98	17.80	**2.02E-09**	**Staurosporine**
**CDK14/cyclin Y (PFTK1)**	74.48	72.89	**1.33E-07**	**Staurosporine**
**CDK16/cyclin Y (PCTAIRE)**	76.57	76.42	**3.18E-08**	**Staurosporine**
**CDK17/cyclin Y (PCTK2)**	54.17	52.31	**1.03E-08**	**Staurosporine**
**CDK18/cyclin Y (PCTK3)**	41.31	36.57	**1.24E-08**	**Staurosporine**
**CDK19/cyclin C**	37.92	37.25	**1.83E-07**	**Staurosporine**
**CDK2/cyclin A**	5.14	4.94	**1.30E-09**	**Staurosporine**
**CDK2/cyclin A1**	7.95	7.85	**4.15E-09**	**Staurosporine**
**CDK2/CYCLIN E**	7.89	5.60	**2.27E-09**	**Staurosporine**
**CDK2/cyclin E2**	11.76	11.44	**3.04E-09**	**Staurosporine**
**CDK2/cyclin O**	4.97	4.80	**2.40E-09**	**Staurosporine**
**CDK3/cyclin E**	13.87	12.85	**2.64E-09**	**Staurosporine**
**CDK3/cyclin E2**	22.31	20.66	**4.31E-09**	**Staurosporine**
**CDK4/cyclin D1**	68.81	68.11	**6.48E-09**	**Staurosporine**
**CDK4/cyclin D3**	85.64	84.60	**1.71E-08**	**Staurosporine**
**CDK5/P25**	7.17	6.97	**1.99E-09**	**Staurosporine**
**CDK5/p35**	8.78	8.70	**1.95E-09**	**Staurosporine**
**CDK6/cyclin D1**	62.97	61.17	**3.56E-09**	**Staurosporine**
**CDK6/cyclin D3**	56.76	54.89	**9.33E-09**	**Staurosporine**
**CDK7/cyclin H**	65.58	65.37	**2.77E-08**	**Staurosporine**
**CDK8/cyclin C**	10.10	10.03	**5.22E-10**	**Staurosporine**
**CDK9/cyclin K**	11.43	11.18	**2.40E-08**	**Staurosporine**
**CDK9/cyclin T1**	13.12	13.12	**5.90E-09**	**Staurosporine**
**CDK9/cyclin T2**	8.55	8.32	**2.15E-09**	**Staurosporine**
**CHK1**	4.66	4.46	**1.81E-10**	**Staurosporine**
**CHK2**	25.42	25.13	**5.48E-09**	**Staurosporine**
**CK1a1**	98.34	97.55	**4.02E-06**	**Staurosporine**
**CK1a1L**	82.84	78.55	**7.11E-07**	**Staurosporine**
**CK1d**	106.16	100.57	**1.49E-07**	**D4476**
**CK1epsilon**	38.82	38.79	**2.61E-07**	**D4476**
**CK1g1**	95.86	92.37	**8.27E-06**	**Staurosporine**
**CK1g2**	98.52	93.32	**1.47E-06**	**Staurosporine**
**CK1g3**	97.22	95.03	**4.61E-06**	**Staurosporine**
**CK2a**	71.37	70.67	**2.67E-07**	**GW5074**
**CK2a2**	44.65	43.00	**1.41E-06**	**Staurosporine**
**CLK1**	4.05	3.79	**1.14E-08**	**Staurosporine**

**CLK2**	4.13	3.10	**3.46E-09**	**Staurosporine**
**CLK3**	35.40	34.93	**1.17E-06**	**Staurosporine**
**CLK4**	10.44	10.10	**5.24E-08**	**Staurosporine**
**COT1/MAP3K8**	109.68	103.54	**7.47E-06**	**Ro-31-8220**
**CSK**	2.95	2.41	**2.45E-08**	**Staurosporine**
**CTK/MATK**	24.61	24.07	**1.49E-07**	**Staurosporine**
**DAPK1**	13.62	13.55	**2.48E-08**	**Staurosporine**
**DAPK2**	11.32	10.10	**5.60E-09**	**Staurosporine**
**DCAMKL1**	83.72	80.77	**7.10E-08**	**Staurosporine**
**DCAMKL2**	87.69	86.26	**1.79E-08**	**Staurosporine**
**DDR1**	0.98	0.67	**7.07E-09**	**Staurosporine**
**DDR2**	1.67	1.45	**7.62E-10**	**Staurosporine**
**DMPK**	76.55	73.32	**1.91E-08**	**Staurosporine**
**DMPK2**	10.86	9.78	**2.92E-10**	**Staurosporine**
**DRAK1/STK17A**	18.20	17.55	**2.89E-08**	**Staurosporine**
**DYRK1/DYRK1A**	2.33	1.92	**4.31E-09**	**Staurosporine**
**DYRK1B**	0.41	0.35	**8.42E-10**	**Staurosporine**
**DYRK2**	9.12	8.90	**1.63E-07**	**Staurosporine**
**DYRK3**	28.20	27.70	**4.48E-08**	**Staurosporine**
**DYRK4**	92.01	91.31	**4.12E-06**	**GW5074**
**EGFR**	17.39	16.47	**6.38E-08**	**Staurosporine**
**EPHA1**	31.47	30.84	**1.59E-07**	**Staurosporine**
**EPHA2**	33.51	31.99	**8.05E-08**	**Staurosporine**
**EPHA3**	37.88	37.82	**9.15E-08**	**Staurosporine**
**EPHA4**	30.47	28.44	**1.15E-08**	**Staurosporine**
**EPHA5**	52.72	52.25	**1.26E-08**	**Staurosporine**
**EPHA6**	34.56	33.33	**3.82E-08**	**Staurosporine**
**EPHA7**	6.91	6.08	**8.59E-08**	**Staurosporine**
**EPHA8**	70.10	69.23	**8.16E-08**	**Staurosporine**
**EPHB1**	1.91	1.62	**3.21E-08**	**Staurosporine**
**EPHB2**	28.50	27.11	**7.34E-08**	**Staurosporine**
**EPHB3**	28.89	28.88	**5.97E-07**	**Staurosporine**
**EPHB4**	19.38	18.92	**2.22E-07**	**Staurosporine**
**ERBB2/HER2**	35.89	34.05	**1.15E-07**	**Staurosporine**
**ERBB4/HER4**	25.01	24.80	**2.25E-07**	**Staurosporine**
**ERK1**	91.79	90.38	**2.42E-09**	**SCH772984**
**ERK2/MAPK1**	75.35	74.09	**4.42E-10**	**SCH772984**
**ERK5/MAPK7**	84.47	81.71	**6.77E-06**	**Staurosporine**
**ERK7/MAPK15**	27.94	27.60	**1.80E-08**	**Staurosporine**
**ERN1/IRE1**	8.49	8.17	**8.11E-08**	**Staurosporine**
**ERN2/IRE2**	6.47	5.93	**3.98E-08**	**Staurosporine**
**FAK/PTK2**	15.52	13.59	**2.29E-08**	**Staurosporine**
**FER**	0.37	0.26	**9.21E-10**	**Staurosporine**
**FES/FPS**	2.77	2.56	**2.16E-09**	**Staurosporine**
**FGFR1**	6.31	6.17	**7.30E-09**	**Staurosporine**
**FGFR2**	2.58	2.54	**4.02E-09**	**Staurosporine**
**FGFR3**	4.66	4.46	**1.36E-08**	**Staurosporine**
**FGFR4**	4.31	4.30	**5.54E-08**	**Staurosporine**

**FGR**	1.68	1.11	**8.33E-10**	**Staurosporine**
**FLT1/VEGFR1**	2.92	2.85	**3.66E-09**	**Staurosporine**
**FLT3**	4.55	4.52	**1.27E-09**	**Staurosporine**
**FLT4/VEGFR3**	1.97	1.86	**3.31E-09**	**Staurosporine**
**FMS**	0.92	0.52	**1.50E-09**	**Staurosporine**
**FRK/PTK5**	4.49	4.46	**1.15E-08**	**Staurosporine**
**FYN**	2.59	2.44	**9.16E-10**	**Staurosporine**
**GCK/MAP4K2**	4.74	4.56	**3.41E-10**	**Staurosporine**
**GLK/MAP4K3**	0.96	0.93	**1.85E-10**	**Staurosporine**
**GRK1**	4.70	4.55	**4.67E-08**	**Staurosporine**
**GRK2**	29.46	28.58	**1.08E-06**	**Staurosporine**
**GRK3**	46.88	46.87	**2.32E-06**	**Staurosporine**
**GRK4**	19.80	19.80	**1.40E-07**	**Staurosporine**
**GRK5**	7.35	7.04	**5.30E-08**	**Staurosporine**
**GRK6**	8.36	8.11	**6.70E-08**	**Staurosporine**
**GRK7**	7.19	6.73	**9.93E-09**	**Staurosporine**
**GSK3a**	2.72	2.26	**3.94E-09**	**Staurosporine**
**GSK3b**	2.85	2.83	**7.03E-09**	**Staurosporine**
**Haspin**	49.47	49.36	**1.61E-08**	**Staurosporine**
**HCK**	7.23	6.46	**3.68E-09**	**Staurosporine**
**HGK/MAP4K4**	0.44	-0.12	**4.86E-10**	**Staurosporine**
**HIPK1**	36.35	33.68	**6.33E-07**	**Ro-31-8220**
**HIPK2**	17.52	17.11	**8.62E-07**	**Staurosporine**
**HIPK3**	14.49	13.73	**8.01E-07**	**Staurosporine**
**HIPK4**	10.70	10.40	**3.79E-07**	**Staurosporine**
**HPK1/MAP4K1**	2.66	2.01	**4.22E-08**	**Ro-31-8220**
**IGF1R**	6.72	6.42	**2.49E-08**	**Staurosporine**
**IKKa/CHUK**	31.57	30.89	**1.06E-07**	**Staurosporine**
**IKKb/IKBKB**	33.35	31.51	**2.57E-07**	**Staurosporine**
**IKKe/IKBKE**	1.47	1.28	**1.80E-10**	**Staurosporine**
**IR**	15.79	15.41	**2.41E-08**	**Staurosporine**
**IRAK1**	3.57	3.16	**5.40E-08**	**Staurosporine**
**IRAK4**	6.48	6.41	**3.45E-09**	**Staurosporine**
**IRR/INSRR**	4.68	3.78	**1.36E-08**	**Staurosporine**
**ITK**	4.12	3.38	**1.05E-08**	**Staurosporine**
**JAK1**	-0.15	-0.64	**6.48E-10**	**Staurosporine**
**JAK2**	1.13	0.89	**2.16E-10**	**Staurosporine**
**JAK3**	0.97	0.84	**1.46E-10**	**Staurosporine**
**JNK1**	24.84	24.80	**3.97E-07**	**Staurosporine**
**JNK2**	42.32	41.78	**1.23E-06**	**Staurosporine**
**JNK3**	23.55	23.39	**3.66E-08**	**JNKi VIII**
**KDR/VEGFR2**	7.22	6.16	**2.19E-08**	**Staurosporine**
**KHS/MAP4K5**	5.19	5.14	**2.44E-10**	**Staurosporine**
**KSR1**	90.75	86.36	**6.62E-06**	**Staurosporine**
**KSR2**	107.87	107.01	**6.83E-06**	**Staurosporine**
**LATS1**	9.89	9.79	**1.82E-08**	**Staurosporine**
**LATS2**	6.54	6.32	**7.87E-09**	**Staurosporine**
**LCK**	3.41	2.99	**2.35E-09**	**Staurosporine**

**LCK2/ICK**	4.57	4.44	**1.32E-08**	**Staurosporine**
**LIMK1**	0.88	0.77	**1.27E-09**	**Staurosporine**
**LIMK2**	67.42	66.53	**7.48E-08**	**Staurosporine**
**LKB1**	37.96	37.39	**5.16E-08**	**Staurosporine**
**LOK/STK10**	3.02	2.86	**4.81E-08**	**Ro-31-8220**
**LRRK2**	1.69	1.00	**7.30E-09**	**Staurosporine**
**LYN**	2.50	1.83	**6.59E-10**	**Staurosporine**
**LYN B**	2.95	2.48	**3.59E-09**	**Staurosporine**
**MAK**	13.19	12.70	**1.24E-08**	**Staurosporine**
**MAPKAPK2**	69.90	67.54	**1.41E-07**	**Staurosporine**
**MAPKAPK3**	87.53	85.18	**5.82E-06**	**Staurosporine**
**MAPKAPK5/PRAK**	85.17	84.10	**3.01E-07**	**Staurosporine**
**MARK1**	1.18	1.16	**1.22E-10**	**Staurosporine**
**MARK2/PAR-1Ba**	2.38	2.21	**6.75E-11**	**Staurosporine**
**MARK3**	1.80	1.76	**2.48E-10**	**Staurosporine**
**MARK4**	0.77	0.72	**5.41E-10**	**Staurosporine**
**MAST3**	106.09	104.37	**6.25E-07**	**Staurosporine**
**MASTL**	33.79	33.66	**4.46E-08**	**Staurosporine**
**MEK1**	76.66	75.35	**5.41E-08**	**Staurosporine**
**MEK2**	67.99	66.36	**5.15E-08**	**Staurosporine**
**MEK3**	49.31	47.29	**1.10E-08**	**Staurosporine**
**MEK5**	40.44	40.06	**3.29E-08**	**Staurosporine**
**MEKK1**	66.97	65.72	**7.69E-07**	**Staurosporine**
**MEKK2**	8.32	8.26	**4.15E-08**	**Staurosporine**
**MEKK3**	6.32	5.56	**2.49E-08**	**Staurosporine**
**MEKK6**	110.20	107.81	**5.29E-07**	**Staurosporine**
**MELK**	1.43	1.31	**1.17E-09**	**Staurosporine**
**MINK/MINK1**	-1.70	-1.95	**7.78E-10**	**Staurosporine**
**MKK4**	99.00	97.17	**1.86E-06**	**Staurosporine**
**MKK6**	59.69	57.24	**1.02E-08**	**Staurosporine**
**MKK7**	96.21	94.42	**2.75E-06**	**Staurosporine**
**MLCK/MYLK**	15.47	14.41	**2.13E-08**	**Staurosporine**
**MLCK2/MYLK2**	27.32	24.42	**3.22E-08**	**Staurosporine**
**MLK1/MAP3K9**	4.06	4.01	**4.67E-10**	**Staurosporine**
**MLK2/MAP3K10**	1.86	1.47	**3.12E-09**	**Staurosporine**
**MLK3/MAP3K11**	-0.81	-1.50	**2.12E-09**	**Staurosporine**
**MLK4**	0.87	0.85	**1.56E-06**	**Staurosporine**
**MNK1**	42.65	41.13	**1.06E-07**	**Staurosporine**
**MNK2**	4.79	4.76	**1.35E-08**	**Staurosporine**
**MRCKa/CDC42BPA**	71.26	68.39	**4.63E-09**	**Staurosporine**
**MRCKb/CDC42BPB**	75.84	73.82	**3.81E-09**	**Staurosporine**
**MSK1/RPS6KA5**	1.53	1.49	**9.57E-10**	**Staurosporine**
**MSK2/RPS6KA4**	2.11	1.99	**2.93E-09**	**Staurosporine**
**MSSK1/STK23**	67.66	67.63	**1.31E-06**	**Staurosporine**
**MST1/STK4**	4.33	3.39	**1.56E-09**	**Staurosporine**
**MST2/STK3**	3.52	3.40	**1.97E-09**	**Staurosporine**
**MST3/STK24**	16.65	15.83	**1.25E-08**	**Staurosporine**
**MST4**	1.35	1.22	**4.52E-09**	**Staurosporine**

**MUSK**	3.33	2.02	**4.56E-09**	**Staurosporine**
**MYLK3**	67.58	64.65	**8.66E-08**	**Staurosporine**
**MYLK4**	6.48	5.38	**3.97E-08**	**Staurosporine**
**MYO3A**	6.46	5.96	**2.63E-08**	**Staurosporine**
**MYO3b**	5.12	4.81	**9.47E-09**	**Staurosporine**
**NEK1**	5.88	5.78	**1.90E-08**	**Staurosporine**
**NEK11**	52.04	49.43	**1.23E-06**	**Staurosporine**
**NEK2**	18.82	18.42	**2.31E-07**	**Staurosporine**
**NEK3**	87.31	85.95	**5.46E-08**	**JNK-IN-7**
**NEK4**	6.24	5.73	**1.48E-07**	**Staurosporine**
**NEK5**	6.56	6.22	**3.04E-08**	**Staurosporine**
**NEK6**	89.04	87.72	**9.32E-06**	**PKR Inhibitor**
**NEK7**	88.55	82.08	**8.35E-06**	**PKR Inhibitor**
**NEK9**	2.81	2.70	**1.28E-07**	**Staurosporine**
**NIM1**	46.64	46.56	**1.49E-07**	**Staurosporine**
**NLK**	72.95	68.44	**1.32E-07**	**Staurosporine**
**OSR1/OXSR1**	54.24	53.02	**9.91E-08**	**Staurosporine**
**P38a/MAPK14**	101.68	100.67	**2.18E-08**	**SB202190**
**P38b/MAPK11**	84.51	83.74	**3.20E-08**	**SB202190**
**P38d/MAPK13**	74.74	69.92	**2.04E-07**	**Staurosporine**
**P38g**	30.29	29.80	**1.58E-07**	**Staurosporine**
**p70S6K/RPS6KB1**	5.46	4.80	**3.34E-10**	**Staurosporine**
**p70S6Kb/RPS6KB2**	3.38	3.26	**1.56E-09**	**Staurosporine**
**PAK1**	8.08	7.21	**3.31E-10**	**Staurosporine**
**PAK3**	6.48	6.27	**1.61E-10**	**Staurosporine**
**PAK4**	7.78	7.24	**2.86E-09**	**Staurosporine**
**PAK5**	7.72	7.41	**4.99E-09**	**Staurosporine**
**PAK6**	9.79	8.98	**5.52E-09**	**Staurosporine**
**PASK**	41.61	40.24	**1.54E-08**	**Staurosporine**
**PBK/TOPK**	69.77	67.76	**1.82E-07**	**Staurosporine**
**PDGFRa**	6.05	5.54	**1.62E-09**	**Staurosporine**
**PDGFRb**	1.90	1.79	**6.91E-09**	**Staurosporine**
**PDK1/PDPK1**	4.24	4.20	**1.16E-09**	**Staurosporine**
**PHKg1**	3.74	3.44	**9.56E-10**	**Staurosporine**
**PHKg2**	18.37	17.90	**1.62E-09**	**Staurosporine**
**PIM1**	0.88	0.79	**2.15E-09**	**Staurosporine**
**PIM2**	21.27	20.94	**1.78E-08**	**Staurosporine**
**PIM3**	2.51	2.18	**1.98E-10**	**Staurosporine**
**PKA**	6.32	6.28	**2.65E-09**	**Staurosporine**
**PKAcb**	1.62	1.23	**7.73E-10**	**Staurosporine**
**PKAcg**	2.07	2.04	**2.38E-09**	**Staurosporine**
**PKCa**	0.86	0.84	**3.85E-10**	**Staurosporine**
**PKCb1**	2.83	2.30	**6.82E-09**	**Staurosporine**
**PKCb2**	1.71	1.66	**4.28E-09**	**Staurosporine**
**PKCd**	8.64	7.89	**2.17E-10**	**Staurosporine**
**PKCepsilon**	1.11	0.77	**2.90E-10**	**Staurosporine**
**PKCeta**	1.78	0.42	**3.54E-10**	**Staurosporine**
**PKCg**	1.77	1.53	**1.57E-10**	**Staurosporine**

**PKCIOTA**	31.54	30.93	**2.26E-08**	**Staurosporine**
**PKCmu/PRKD1**	30.28	28.32	**2.38E-09**	**Staurosporine**
**PKCnu/PRKD3**	11.43	11.12	**2.32E-09**	**Staurosporine**
**PKCtheta**	7.08	6.89	**2.55E-09**	**Staurosporine**
**PKCzeta**	55.85	55.80	**6.22E-08**	**Staurosporine**
**PKD2/PRKD2**	11.93	11.75	**1.51E-09**	**Staurosporine**
**PKG1a**	7.02	6.41	**1.46E-09**	**Staurosporine**
**PKG1b**	13.87	12.45	**5.59E-09**	**Staurosporine**
**PKG2/PRKG2**	1.99	1.33	**2.70E-09**	**Staurosporine**
**PKN1/PRK1**	2.22	2.15	**1.39E-09**	**Staurosporine**
**PKN2/PRK2**	6.62	6.60	**3.97E-09**	**Staurosporine**
**PKN3/PRK3**	20.17	14.75	**8.93E-09**	**Staurosporine**
**PLK1**	17.14	17.05	**1.56E-07**	**Staurosporine**
**PLK2**	27.38	26.59	**2.22E-07**	**Staurosporine**
**PLK3**	98.18	93.76	**2.62E-09**	**BI2536**
**PLK4/SAK**	6.73	6.38	**1.44E-08**	**Staurosporine**
**PRKX**	9.56	9.38	**9.62E-10**	**Staurosporine**
**PYK2**	2.40	2.27	**1.13E-08**	**Staurosporine**
**RAF1**	111.46	109.90	**7.01E-09**	**GW5074**
**RET**	2.10	2.10	**2.82E-09**	**Staurosporine**
**RIPK2**	41.72	40.54	**1.16E-07**	**Staurosporine**
**RIPK4**	99.11	98.47	**5.11E-07**	**Staurosporine**
**RIPK5**	22.66	22.32	**2.88E-08**	**Staurosporine**
**ROCK1**	26.20	25.74	**1.37E-09**	**Staurosporine**
**ROCK2**	12.69	12.53	**8.88E-10**	**Staurosporine**
**RON/MST1R**	25.08	25.03	**1.39E-07**	**Staurosporine**
**ROS/ROS1**	4.57	4.19	**2.52E-10**	**Staurosporine**
**RSK1**	3.27	2.95	**1.61E-10**	**Staurosporine**
**RSK2**	1.69	1.58	**4.93E-10**	**Staurosporine**
**RSK3**	3.38	3.24	**1.52E-10**	**Staurosporine**
**RSK4**	6.64	6.54	**1.29E-10**	**Staurosporine**
**SBK1**	78.83	76.94	**6.01E-08**	**Staurosporine**
**SGK1**	13.38	12.71	**1.56E-08**	**Staurosporine**
**SGK2**	22.64	22.07	**9.55E-09**	**Staurosporine**
**SGK3/SGKL**	59.57	58.53	**1.19E-07**	**Staurosporine**
**SIK1**	1.00	0.64	**1.60E-09**	**Staurosporine**
**SIK2**	1.49	1.17	**4.95E-10**	**Staurosporine**
**SIK3**	0.42	0.08	**1.53E-09**	**Staurosporine**
**SLK/STK2**	6.42	6.42	**2.31E-08**	**Staurosporine**
**SNARK/NUAK2**	2.16	1.92	**2.73E-09**	**Staurosporine**
**SNRK**	72.54	72.07	**2.86E-08**	**Staurosporine**
**SRMS**	90.96	90.39	**7.05E-06**	**Staurosporine**
**SRPK1**	9.17	8.28	**2.21E-08**	**Staurosporine**
**SRPK2**	8.02	7.62	**1.40E-07**	**Staurosporine**
**SSTK/TSSK6**	95.67	94.64	**2.17E-07**	**Staurosporine**
**STK16**	10.21	9.79	**2.51E-07**	**Staurosporine**
**STK21/CIT**	81.66	81.15	**1.51E-07**	**Staurosporine**
**STK22D/TSSK1**	2.71	2.56	**5.31E-11**	**Staurosporine**

**STK25/YSK1**	4.17	3.00	**9.64E-09**	**Staurosporine**
**STK32B/YANK2**	0.96	0.91	**2.90E-08**	**Staurosporine**
**STK32C/YANK3**	20.52	20.31	**2.42E-07**	**Staurosporine**
**STK33**	9.17	8.22	**3.41E-08**	**Staurosporine**
**STK38/NDR1**	9.08	8.29	**5.74E-10**	**Staurosporine**
**STK38L/NDR2**	22.67	22.41	**3.00E-09**	**Staurosporine**
**STK39/STLK3**	19.65	18.27	**1.90E-08**	**Staurosporine**
**SYK**	0.87	0.78	**9.24E-10**	**Staurosporine**
**TAK1**	7.20	6.81	**6.09E-08**	**Staurosporine**
**TAOK1**	6.83	6.33	**2.05E-09**	**Staurosporine**
**TAOK2/TAO1**	36.34	35.06	**6.77E-09**	**Staurosporine**
**TAOK3/JIK**	12.84	12.63	**7.23E-09**	**Staurosporine**
**TBK1**	2.01	1.90	**1.00E-09**	**Staurosporine**
**TEC**	19.95	19.75	**6.93E-08**	**Staurosporine**
**TESK1**	82.95	82.57	**3.60E-07**	**Staurosporine**
**TESK2**	91.15	89.59	**5.55E-06**	**Staurosporine**
**TGFBR2**	110.71	108.40	**1.09E-07**	**LDN193189**
**TIE2/TEK**	7.56	7.23	**6.00E-08**	**Staurosporine**
**TLK1**	26.05	25.57	**2.22E-08**	**Staurosporine**
**TLK2**	67.01	66.39	**3.94E-09**	**Staurosporine**
**TNIK**	1.22	1.13	**6.79E-10**	**Staurosporine**
**TNK1**	3.88	2.88	**5.28E-09**	**Staurosporine**
**TRKA**	-2.22	-3.31	**8.72E-09**	**Staurosporine**
**TRKB**	1.79	1.73	**3.73E-10**	**Staurosporine**
**TRKC**	0.34	0.18	**1.17E-10**	**Staurosporine**
**TSSK2**	21.20	21.04	**1.15E-08**	**Staurosporine**
**TSSK3/STK22C**	62.20	61.46	**8.16E-09**	**Staurosporine**
**TTBK1**	67.65	66.32	**6.89E-06**	**SB202190**
**TTBK2**	95.09	94.91	**4.31E-06**	**SB202190**
**TXK**	3.64	3.62	**3.58E-08**	**Staurosporine**
**TYK1/LTK**	7.18	6.71	**2.38E-08**	**Staurosporine**
**TYK2**	6.35	5.86	**2.50E-10**	**Staurosporine**
**TYRO3/SKY**	23.63	23.35	**6.56E-09**	**Staurosporine**
**ULK1**	13.58	13.46	**9.04E-09**	**Staurosporine**
**ULK2**	13.64	13.54	**3.44E-09**	**Staurosporine**
**ULK3**	14.16	11.94	**3.38E-09**	**Staurosporine**
**VRK1**	81.57	79.87	**7.34E-07**	**Ro-31-8220**
**VRK2**	81.76	81.67	**1.70E-05**	**Ro-31-8220**
**WEE1**	44.25	43.28	**3.59E-08**	**Wee-1 Inhibitor**
**WNK1**	109.55	107.75	**4.83E-05**	**Staurosporine**
**WNK2**	71.92	69.29	**4.74E-06**	**Staurosporine**
**WNK3**	66.36	64.44	**1.49E-06**	**Wee-1 Inhibitor**
**YES/YES1**	1.40	1.32	**2.01E-09**	**Staurosporine**
**YSK4/MAP3K19**	3.40	3.38	**8.57E-09**	**Staurosporine**
**ZAK/MLTK**	82.68	82.32	**1.89E-06**	**GW5074**
**ZAP70**	12.98	12.64	**1.94E-08**	**Staurosporine**
**ZIPK/DAPK3**	6.48	5.05	**4.17E-09**	**Staurosporine**

**AMPK (A1/B2/G2)**	-0.06	-0.34	**6.35E-08**	**Ro-31-8220**
**AMPK (A1/B2/G3)**	1.13	1.09	**1.15E-07**	**Ro-31-8220**
**AMPK (A2/B1/G2)**	0.66	0.09	**1.06E-07**	**Ro-31-8220**
**AMPK (A2/B1/G3)**	0.67	0.59	**2.96E-07**	**Ro-31-8220**
**AMPK(A1/B1/G1)**	1.14	1.14	**6.68E-08**	**Ro-31-8220**
**AMPK(A1/B1/G2)**	1.46	1.23	**7.68E-08**	**Ro-31-8220**
**AMPK(A1/B1/G3)**	0.22	0.07	**1.79E-07**	**Ro-31-8220**
**AMPK(A1/B2/G1)**	0.73	0.57	**1.16E-07**	**Ro-31-8220**
**AMPK(A2/B1/G1)**	0.76	0.64	**2.12E-07**	**Ro-31-8220**
**AMPK(A2/B2/G1)**	0.34	0.23	**9.78E-08**	**Ro-31-8220**
**AMPK(A2/B2/G2)**	0.73	0.63	**1.00E-07**	**Ro-31-8220**
**AMPK(A2/B2/G3)**	0.63	0.61	**1.86E-07**	**Ro-31-8220**
**DNA-PK**	98.20	95.76	**1.38E-08**	**PI-103**
**EEF2K**	99.51	97.05	**7.40E-06**	**NH125**
**EIF2AK1**	82.48	81.15	**9.62E-08**	**GSK-2606414**
**EIF2AK2**	9.89	9.34	**5.80E-08**	**Staurosporine**
**EIF2AK3**	61.60	60.18	**2.76E-09**	**GSK-2606414**
**EIF2AK4**	9.50	9.39	**7.51E-07**	**Staurosporine**
**mTOR/FRAP1**	111.59	107.76	**5.01E-08**	**PI-103**
**PDK1/PDHK1**	97.24	96.82	**8.63E-06**	**GW5074**
**PDK2/PDHK2**	95.46	93.95	**5.05E-06**	**GW5074**
**PDK3/PDHK3**	96.40	94.81	**2.57E-06**	**GW5074**
**PDK4/PDHK4**	93.19	92.98	**1.89E-06**	**GW5074**
**TRPM7/CHAK1**	91.59	91.48	**2.34E-05**	**NH125**
