# Supplementary material for: Cell-impermeable staurosporine analog targets extracellular kinases to inhibit HSV and SARS-CoV-2
Source: Commun Biol. 2022 Oct 16;5:1096. doi: 10.1038/s42003-022-04067-4 (PMC9569420; doi:10.1038/s42003-022-04067-4)
Supplement: Supplementary file 4 — Supplementary Data 2 [file 42003_2022_4067_MOESM4_ESM.pdf]

Fig 1b 24 h

	Media	0.1%	0.5%	1%	0.1µM	1µM	10µM	50µM	100µM	0.1µM	1µM	10µM	50µM	100µM
	1.7153	1.4935	1.5377	1.4357	1.6165	1.5244	1.632	1.4999	1.632	1.2348	0.5819	0.4002	0.4492	0.4385
	1.851	2.0673	1.6894	1.4458	1.4879	1.2545	1.7765	1.8351	1.7765	1.4306	0.7217	0.4124	0.5219	0.5067
	1.6228	1.3967	1.6785	1.435	1.4117	1.4999	1.2294	1.6165	1.0397	1.2867	0.9947	0.4023	0.4937	0.5605
	1.5821	1.8431	1.6882	1.4292	1.7957	1.8351	1.4658	1.4879	1.4289	1.3154	0.8163	0.4178	0.5097	0.4661

Fig 1b 72 h

	Media	0.1%	0.5%	1%	0.1µM	1µM	10µM	50µM	100µM	0.1µM	1µM	10µM	50µM	100µM
	1.9161	1.6482	2.2246	1.9015	1.9672	2.3853	1.5817	1.6711	0.6851	1.1476	0.4328	0.338	0.3395	0.296
	2.4476	1.9313	2.3661	1.8852	2.1091	2.3118	2.3121	1.6282	0.727	1.088	0.3057	0.3413	0.3136	0.3252
	2.034	1.7259	2.1041	1.9695	1.6205	2.4626	1.3143	1.5817	0.7321	1.254	0.427	0.3855	0.3357	0.3057
	1.8777	2.4284	2.1328	1.5204	2.4775	2.6157	1.8471	1.8471	0.7205	1.088	0.4657	0.3533	0.3014	0.3357

Figure 1c

HaCat	Control buffer				CIMSS				Staurosporine			
24 h	106	87	95.5	93.86	123	93.2	114	107	18.5	22.8	20.1	18.03
72 h	125	128	112		108	87.6	102	98	0	0	0	0
120 h	93.5	94.6	83.2	90	98	98	88.4	84	0	0	0	0

Figure 1d

Primary vaginal

DMSO	0 µM CIMS	0 µM CIMS	1 µM Staur	0 µM Stauro
99	99.5	93	39.5	42.8
101	102	91.8	67.5	18.8

Fig 2b

	**Activated caspase positive**			**SYTOX green positive**		
DMSO	6	5.2		3	0	
CIMSS	0	0	5	0	0	2.7
Stauro	100	75	87.5	88.8	91.6	75

Fig. 3a

HaCat

DMSO	0.1	1	10	50
101.8	74	70	26.8	30.9
98.66	73.4	69	30.9	30.2
108	64	45	10	14
101.3	57	52	14.36	25

Primary vaginal cells

DMSO	0.1	1	10	50
101.8	74	70	26.8	30.9
98.66	73.4	69	30.9	30.2
108	64	45	10	14
101.3	57	52	14.36	25

Fig. 3b

dose	DMSO		10 µM CIMSS		DMSO,CaSki		CIMSS,CaSki	
0	8	6	4	5	20	24	2	7
0.5	154	159	27	13	184	136	49	51
1	148	149	11	17	148	149	48	46
1.5	161	172	16	19	161	172	36	47
2	165	207	19	11	172	176	52	37

Fig 4b

**Time min**	DMSO	**CIMSS**	HSV-2	SV-2+CIMSS
1	35.28571	30.13793	62.18182	42.29032
2	20.83871	38.92683	141	147.8919
3	31.81395	48.85714	101.3333	79.8
4	21.27508	46.76923	171.01	106.2273
5	18	48.85714	162.8571	79.8
6	18.83898	42.75	171	72.34615
7	19.26249	25.95536	160.5042	68.83019
8	11.02493	43.93451	147.8919	62.18182
9	11.6513	44.35333	179.6364	65.44444
10	14.22086	40.26252	198.5806	47.5
11	11.6513	35.28571	162.8571	50.23729
12	9.546798	36.21562	273.6	50.23729
13	9.303835	38.92683	167.686	65.44444
14	7.276596	33.52941	179.6364	55.97018
15	12.41129	40.81481	183.6959	62.18182
16	12.79474	42.75	201.6352	59.03571
17	11.27473	30.13793	197.1753	62.18182
18	16.08299	35.28571	185.3513	61.09939
19	13.18053	38.92683	162.0684	67.1215
20	11.02493	38.92683	224.8112	77.50198
21	18.83898	33.52941	188.8125	92.17021
22	25.33333	37.08434	220.1379	92.17021
23	17.3089	37.08434	220.1379	59.03571
24	21.71429	37.08434	196.2786	76
25	19.6887	38.92683	268.3994	83.71475
26	28.5	35.28571	196.2786	100.8559
27	20.83871	38.92683	196.2786	100.8559
28	31.9837	33.52941	268.3994	87.875
29	30.9711	45.31902	234.5612	77.50198
30	37.81598	43.73585	234.5612	68.83019

Fig 4 c

3 minutes

DMSO	DMSO+HSV	CIMSS	CIMSS+HSV
29.30814	53.74	38.88	42.20119
40.72333	81.1143	30.45333	89.92
29.31279	84.49	39.3074	74.0688
29.31	101.105	44.7233	89.99

1 hour

DMSO	HSV-2	CIMSS	SV-2+CIMSS
75.05473	202.6667	90.66786	46.11236
61.38462	253.7419	95.94475	79.54839
106.9302	470.6154	99.48315	108.2222
114	253.7419	102.73	119.6066

Fig. 6c siAkt

siControl	siAkt1		siControl	siPDPK1
93.4	19.56		100.6	32.8
106.5	23.9		99.3	34.26
96.77	25.8		96.77	6.45
103	21.5		103	4.3

Fig. 7a

		Control buffer				CIMSS		
VSV-G	100	79	96	100	96.5	100	96	100
VSV-S	88.3	87.98			15.7	14.3		
VSV-EBOV	71.6	87			82.4	68		

Fig 7c

0	0.1	1	10	50	100	0	0.1	1	10	50	100	0	0.1	1	10	50	100
116	66.7	61.8	55.7	30	28.7	114.2	79.7	77.65	61.7	41.48	12.76	92.1	39.3	36.9	28	28	25
83.6	69	65.4	58.1	26.9	26.3	107.1	84	67.02	52.1	42.55	15.95	107	47.1	16.8	34.8	26	24.2
92.18	76.6	38.35	23.4	12.76	2.1	101	70.9	67.4	52.57	42.2	8	109		30.9		16.9	0
107	68.08	29.8	36.1	19.1	10.3	98.92	80.4	81.1	56	40	9.1	90		50.7		22.5	19.7

Fig 7d

Vero						Huh7						Calu-3					
0	0.1	1	10	50	100	0	0.1	1	10	50	100	0	0.1	1	10	50	100
97.7	86.6	77.7	58.3	51.1	36.1	91.3	82.1	87.4	51.6	37.08	23.8	89.7	84.6	28.2	7.6	7.6	7.6
102	75.5	54.4	65.5	46.6	46.6	108.6	86.1	59.6	50.3	29.13	25.16	110	82.05	41.02	17.9	7.6	20
98.6	70.6	48	48	40	29.7	90.3	73	59	59	48	55						
107.5	70.6	75.3	44.6	37	43	109	78.8	69	56	38	42						

Fig 7e 24h

conc. [uM]		infection			viability	
0.1	98.46062	107.6173	93.91948	96.27722	103.2615	100.4613
0.78125	85.80985	65.46244	126.7822	84.73044	82.01673	92.57966
1.5625	94.42832	80.33535	112.8366	85.45482	95.02308	79.37871
3.125	117.6404	65.80843	78.31515	87.56308	97.45569	88.74154
6.25	68.45137	109.6568	117.739	87.4874	88.95778	99.59638
12.5	55.82165	62.24444	57.91311	93.48783	95.45554	94.40682
25	43.89535	23.98594	22.99528	87.93067	93.30404	92.73102
50	18.57264	11.45406	20.69054	87.27117	89.31456	97.15296

72 h

conc. [uM]		infection			viability	
0.1	76.86317	123.2695		109.6641	90.33516	108.5913
0.78125	162.7211	86.9263	67.5887	94.77191	98.60861	93.6809
1.5625	90.30519			110.5187	106.5002	93.2445
3.125	48.24051	144.0635		88.95322	84.08007	95.59016
6.25	73.87688	30.36319	4.01902	97.20848	97.66307	91.62618
12.5	21.36103	1.28361	12.8489	90.13514	84.35282	83.66185
25	2.124058	20.54583	2.28356	80.38883	65.49663	71.82445
50	1.451745	20.56501	1.25226	70.09703	68.89692	78.71596

Fig 9b

VSV					VSV-S			
siPDPK1	siAkt1	siPLCγ1	siFIC-1		siPDPK1	siAkt1	siPLCγ1	siFIC-1
86.3	87.5	87.5	94.4		16.2	23.2	35	75.8
109	91.6	84.7	91.6		11.2	33.5	31	81.9
81	78.6	76	92.5		38	45.3	46.9	84.5
70	80.3	80.3	87.1		24.7	43.2	37.1	88.7
112	122.5		122		34.75	34		95
80	137		107		30.49	29.78		75.7

Fig 9d

		mIgG				Ace2				PtDS				Akt				PDPK1		
VSV-G	75.4	101	122	87.7	94.7	108	119	139	107	126	101	126	125	123	94.7	101	104	110	126	115
VSV-S	117	107	82.9	81.3	58.5	66.7	43.9	47.1	61.8	55	52	55.3	48.7	50	56	61	48.7	45.5	40.6	35.7
