# Supplementary material for: Cell-impermeable staurosporine analog targets extracellular kinases to inhibit HSV and SARS-CoV-2
Source: Commun Biol. 2022 Oct 16;5:1096. doi: 10.1038/s42003-022-04067-4 (PMC9569420; doi:10.1038/s42003-022-04067-4)
Supplement: Supplementary file 5 — Reporting Summary [file 42003_2022_4067_MOESM5_ESM.pdf]

nature portfolio

Corresponding author(s): Betsy C. Herold

Last updated by author(s): Sep 27, 2022

Reporting Summary

Nature Portfolio wishes to improve the reproducibility of the work that we publish. This form provides structure for consistency and transparency in reporting. For further information on Nature Portfolio policies, see our Editorial Policies and the Editorial Policy Checklist.

Statistics

For all statistical analyses, confirm that the following items are present in the figure legend, table legend, main text, or Methods section.

n/a	Confirmed	
☐	☒	The exact sample size (*n*) for each experimental group/condition, given as a discrete number and unit of measurement
☐	☒	A statement on whether measurements were taken from distinct samples or whether the same sample was measured repeatedly
☐	☒	The statistical test(s) used AND whether they are one- or two-sided<br>*Only common tests should be described solely by name; describe more complex techniques in the Methods section.*
☒	☐	A description of all covariates tested
☐	☒	A description of any assumptions or corrections, such as tests of normality and adjustment for multiple comparisons
☐	☒	A full description of the statistical parameters including central tendency (e.g. means) or other basic estimates (e.g. regression coefficient) AND variation (e.g. standard deviation) or associated estimates of uncertainty (e.g. confidence intervals)
☒	☐	For null hypothesis testing, the test statistic (e.g. *F*, *t*, *r*) with confidence intervals, effect sizes, degrees of freedom and *P* value noted<br>*Give P values as exact values whenever suitable.*
☒	☐	For Bayesian analysis, information on the choice of priors and Markov chain Monte Carlo settings
☒	☐	For hierarchical and complex designs, identification of the appropriate level for tests and full reporting of outcomes
☒	☐	Estimates of effect sizes (e.g. Cohen's *d*, Pearson's *r*), indicating how they were calculated

Our web collection on statistics for biologists contains articles on many of the points above.

Software and code

Policy information about availability of computer code

Data collection	No software was used
Data analysis	No software was used

For manuscripts utilizing custom algorithms or software that are central to the research but not yet described in published literature, software must be made available to editors and reviewers. We strongly encourage code deposition in a community repository (e.g. GitHub). See the Nature Portfolio guidelines for submitting code & software for further information.

Data

Policy information about availability of data

All manuscripts must include a data availability statement. This statement should provide the following information, where applicable:

- Accession codes, unique identifiers, or web links for publicly available datasets
- A description of any restrictions on data availability
- For clinical datasets or third party data, please ensure that the statement adheres to our policy

Data supporting the findings of the study are available within the article and in the accompanying Supplementary Data 1 and Supplementary Data 2 files. All other data are available from the corresponding authors upon request.

Human research participants

Policy information about studies involving human research participants and Sex and Gender in Research.

Reporting on sex and gender	*Use the terms sex (biological attribute) and gender (shaped by social and cultural circumstances) carefully in order to avoid confusing both terms. Indicate if findings apply to only one sex or gender; describe whether sex and gender were considered in study design whether sex and/or gender was determined based on self-reporting or assigned and methods used. Provide in the source data disaggregated sex and gender data where this information has been collected, and consent has been obtained for sharing of individual-level data; provide overall numbers in this Reporting Summary. Please state if this information has not been collected. Report sex- and gender-based analyses where performed, justify reasons for lack of sex- and gender-based analysis.*
Population characteristics	*Describe the covariate-relevant population characteristics of the human research participants (e.g. age, genotypic information, past and current diagnosis and treatment categories). If you filled out the behavioural & social sciences study design questions and have nothing to add here, write "See above."*
Recruitment	*Describe how participants were recruited. Outline any potential self-selection bias or other biases that may be present and how these are likely to impact results.*
Ethics oversight	*Identify the organization(s) that approved the study protocol.*

Note that full information on the approval of the study protocol must also be provided in the manuscript.

Field-specific reporting

Please select the one below that is the best fit for your research. If you are not sure, read the appropriate sections before making your selection.

☒ Life sciences ☐ Behavioural & social sciences ☐ Ecological, evolutionary & environmental sciences

For a reference copy of the document with all sections, see nature.com/documents/nr-reporting-summary-flat.pdf

Life sciences study design

All studies must disclose on these points even when the disclosure is negative.

Sample size	Formal sample size calculations are not appropriate for the study but only enzymology studies were done in duplicate all virology experiments were conducted with a minimum of duplicate technical determinations and a minimum of 2 independent experiments. This data is included in the manuscript.
Data exclusions	No exclusions
Replication	All experiments were conducted with a minimum of duplicate measures and two independent experiments.
Randomization	Randomization is not relevant to the study but all experiments included controls treated with the same concentration of DMSO as the drug treatment group.
Blinding	Blinding was not relevant to the study but as noted above all experiments included appropriate controls.

Reporting for specific materials, systems and methods

We require information from authors about some types of materials, experimental systems and methods used in many studies. Here, indicate whether each material, system or method listed is relevant to your study. If you are not sure if a list item applies to your research, read the appropriate section before selecting a response.

Materials & experimental systems

n/a	Involved in the study	
☐	☒	Antibodies
☐	☒	Eukaryotic cell lines
☒	☐	Palaeontology and archaeology
☒	☐	Animals and other organisms
☒	☐	Clinical data
☒	☐	Dual use research of concern

Methods

n/a	Involved in the study	
☒	☐	ChIP-seq
☒	☐	Flow cytometry
☒	☐	MRI-based neuroimaging

Antibodies

Antibodies used	Primary antibodies and dilutions were as follows: mouse anti-PtdS mAb, 1:200 (05-719, Millipore, Upstate Biotechnology, Lake Placid, NY); mouse anti-PLSCR1 mAb, 1:200 (ab24923, Abcam Cambridge, MA), rabbit anti-PLSCR1, 1:500 (NBP1-322588, NOVUS Biologicals, Littleton, CO); mouse anti-PLSCR1 (sc-27779, Santa Cruz Biotechnology); mouse anti-phosphotyrosine mAb (4G10; 05-1050X, Millipore,); rabbit anti-FIC1, 1:500 (sc-134967, Santa Cruz Biotechnology), mouse anti-β-actin mAb, 1:5000 (A-5441, Sigma-Aldrich); rabbit anti-phospho-Akt (Ser-473) mAb, 1:500 (4060T, Cell Signaling Technology, Danvers, MA); rabbit anti-phospho-Akt (Thr-308) mAb, 1:500 (9275, Cell Signaling Technology); rabbit anti-Akt123, 1:1000 (sc-8312, Santa Cruz Biotechnology); rabbit anti-Akt,1:200 (9272S, Cell Signaling); rabbit anti-Akt1, 1:200 (SAB450007, Sigma Aldrich); rabbit anti-pan-Akt (phosphoT308),1:250 (ab38449, Abcam); rabbit anti-PDPK1,1:200 (3062S, Cell Signaling); rabbit anti-pPDPK1(S241), 1:300 (3438S, Cell Signaling); rabbit anti-pPLC[illegible] 1,1:300 (07-506, Upstate); mouse anti-PLC[illegible]1, 1:200 (sc-374467, Santa Cruz Biotechnology); rabbit anti-cleaved caspase 8 (Asp374) (18C8), 1:1000 (9496, Cell Signaling Technology); rabbit anti-cleaved PARP-1 (Asp 214) (D64E10) XP, 1:1000 (5625, Cell Signaling Technology); mouse anti-ACE2, 1:100 (sc-390851, Santa Cruz Biotechnology), mouse anti-TPMRSS2, 1:100 (sc-51572, Santa Cruz Biotechnology), human anti-SARS-CoV2 Spike protein, 1:100 (703973, Invitrogen); mouse anti-HSV gD, 1:100 (HA025, Virusys Corporation, Taneytown, MD); goat anti-HSV VP16, 2 ug/ml (sc-17547, Santa Cruz Biotechnology); mouse anti-histone H1, 2 ug/ml (sc-8030, Santa Cruz Biotechnology); anti-goat IgG, 1:500 (STAR AR122, Bio-Rad), control mouse IgG, 1:100 (sc-2025, Santa Cruz Biotechnology, goat anti-β-actin (Thermo Fisher Scientific), and rabbit anti-GFP, 1:250 (ab32146, Abcam). Annexin V Alexa Fluor 555 was purchased from Thermo Fisher Scientific (A 35108). The secondary antibodies for Western blots were horseradish peroxidase-conjugated goat anti-mouse (170-5047, Bio-Rad, Hercules, CA), goat anti-rabbit (170-5046, Bio-Rad), and donkey anti-goat 1:1000 (sc-2020, Santa Cruz). The secondary antibodies for confocal microscopy were goat anti-mouse Alexa Fluor 350 (A-11045, Invitrogen Molecular Probes), goat anti-mouse Alexa Fluor 555 (A-21147, Thermo Fisher), goat anti mouse Alexa Fluor 488 (A11001, Thermo Fisher) and goat anti-rabbit Alexa Fluor 488 (A-11078, Thermo Fisher) or Alexa Fluor 555 (A-21428, Thermo Fisher). All secondary antibodies were diluted 1:1000.
Validation	All antibodies were obtained commercially and were validated by the indicated company per their website typicallyusing recombinant proteins with Western blots shown in the data sheet. We include lot numbers and concentrations used for each antibody in the methods section of the mspt with controls in each experiment.

Eukaryotic cell lines

Policy information about cell lines and Sex and Gender in Research

Cell line source(s)	Vero, HaCat, CaCo-2, and Calu-3 cells were obtained from American Type Culture Collection, ATCCC, Manassas VA. Huh7 and the derivative Huh-7.5 were obtained from K Chandran and C. Rice labs and are referenced (Blight, K. J., McKeating, J. A. & Rice, C. M. Highly permissive cell lines for subgenomic and genomic hepatitis C virus RNA replication. J Virol 76, 13001-13014, doi:10.1128/jvi.76.24.13001-13014.2002). Primary human vaginal epithelial cells were obtained from ATCC (PCS-480-010).
Authentication	The cell lines were not authenticated
Mycoplasma contamination	All cell lines were tested for Mycoplasma using LookOut Mycoplasma PCR Detection kit (Sigm-Aldrich).
Commonly misidentified lines (See ICLAC register)	No commonly misidentified lines were used.
